# Acute Nicotine Exposure Alters Ventral Tegmental Area Inhibitory Transmission and Promotes Diazepam Consumption

**DOI:** 10.1523/ENEURO.0348-19.2020

**Published:** 2020-03-06

**Authors:** Alexey Ostroumov, Ruthie E. Wittenberg, Blake A. Kimmey, Madison B. Taormina, William M. Holden, Albert T. McHugh, John A. Dani

**Affiliations:** Department of Neuroscience, Mahoney Institute for Neurosciences, Perelman School of Medicine, University of Pennsylvania, Philadelphia, PA 19104

**Keywords:** benzodiazepines, chloride, dopamine, GABA, KCC2, mesolimbic

## Abstract

Nicotine use increases the risk for subsequent abuse of other addictive drugs, but the biological basis underlying this risk remains largely unknown. Interactions between nicotine and other drugs of abuse may arise from nicotine-induced neural adaptations in the mesolimbic dopamine (DA) system, a common pathway for the reinforcing effects of many addictive substances. Previous work identified nicotine-induced neuroadaptations that alter inhibitory transmission in the ventral tegmental area (VTA). Here, we test whether nicotine-induced dysregulation of GABAergic signaling within the VTA increases the vulnerability for benzodiazepine abuse that has been reported in smokers. We demonstrate in rats that nicotine exposure dysregulates diazepam-induced inhibition of VTA GABA neurons and increases diazepam consumption. In VTA GABA neurons, nicotine impaired KCC2-mediated chloride extrusion, depolarized the GABA_A_ reversal potential, and shifted the pharmacological effect of diazepam on GABA neurons from inhibition toward excitation. In parallel, nicotine-related alterations in GABA signaling observed *ex vivo* were associated with enhanced diazepam-induced inhibition of lateral VTA DA neurons *in vivo*. Targeting KCC2 with the agonist CLP290 normalized diazepam-induced effects on VTA GABA transmission and reduced diazepam consumption following nicotine administration to the control level. Together, our results provide insights into midbrain circuit alterations resulting from nicotine exposure that contribute to the abuse of other drugs, such as benzodiazepines.

## Significance Statement

Benzodiazepines, particularly diazepam, are among the most frequently prescribed drugs for their anxiolytic, sedative, and anti-convulsant effects. In general, benzodiazepines have a relatively low abuse liability, yet misuse occurs more frequently among smokers. Pharmacologically, benzodiazepines act on the GABA_A_ receptor (GABA_A_R), enhancing the effect of the inhibitory neurotransmitter. Our work suggests that nicotine increases vulnerability to diazepam use via aberrant inhibitory signaling in the ventral tegmental area (VTA), a principle nucleus of the brain’s reward system. Specifically, we found that nicotine exposure impaired VTA GABA_A_R function, resulting in altered effects of diazepam on dopamine (DA) circuitry and increased diazepam consumption. Furthermore, we identify a novel therapeutic target, which may serve to mitigate excessive diazepam consumption in smoking or vaping individuals.

## Introduction

Nicotine is the main addictive component of tobacco and electronic cigarettes. Epidemiological and animal studies indicate that, along with other harmful effects, nicotine use positively correlates with the intake of other addictive drugs, acting as a gateway for subsequent substance abuse (Kandel, 1975; Boscarino et al., 2010; Huizink et al., 2010; Harrison and McKee, 2011; Levine et al., 2011; Mello and Newman, 2011; Doyon et al., 2013; Kandel and Kandel, 2014; Miech et al., 2016; Thomas et al., 2018). The specific mechanisms by which nicotine increases vulnerability for subsequent addiction to other substances are not fully understood, but likely involve nicotine-induced adaptations in the reward system of the brain. Accumulating evidence indicates that chronic nicotine exposure dysregulates GABAergic signaling in the ventral tegmental area (VTA), a key dopaminergic brain area involved in the rewarding properties of virtually all addictive drugs (Vihavainen et al., 2008b; Li et al., 2014; Buczynski et al., 2016; Thomas et al., 2018). To date, nicotine-induced changes in VTA GABAergic transmission have been shown to influence dopamine (DA) signaling and behavioral responses to alcohol and morphine (Vihavainen et al., 2008a,b; Thomas et al., 2018). We postulate that, in addition to these drugs, nicotine-induced neuroadaptation of VTA inhibitory transmission can modify the action of addictive substances that specifically target GABA_A_ receptors (GABA_A_R).

Among the different classes of GABA_A_R-targeting drugs, benzodiazepine misuse is a growing public health problem in the United States, with prescription rates and overdose mortality spiking in recent years (Bachhuber et al., 2016; Lembke et al., 2018; Maust et al., 2019). Acting through positive allosteric modulation of GABA_A_R, benzodiazepines are regularly prescribed for panic attacks, insomnia, and seizures. Unfortunately, these drugs also retain a liability for abuse and dependence, especially in polydrug users and individuals with comorbid anxiety and depression (Woods et al., 1992; Griffiths and Weerts, 1997; Licata and Rowlett, 2008). Interestingly, benzodiazepine abuse is more likely to occur in smokers (Dunbar et al., 1988; Lekka et al., 1997; Neutel, 2005), and evidence from rodents shows that exposure to nicotine increases the reinforcing properties of benzodiazepines (White, 1989; Irvine et al., 2001; James-Walke et al., 2007). Like nicotine, benzodiazepine reinforcement involves midbrain GABAergic signaling, as genetic manipulation of GABA_A_Rs in the VTA was shown to decrease benzodiazepine consumption (Tan et al., 2010; Tolu et al., 2013). In contrast to DA neurons, VTA GABA neurons are particularly sensitive to benzodiazepines, demonstrating drug-mediated inhibition of neuronal firing (O'Brien and White, 1987; Tan et al., 2010). For these reasons, VTA GABA neurons may represent a critical locus of interaction for the association between nicotine exposure and subsequent benzodiazepine abuse.

To examine basic neural mechanisms linking nicotine to benzodiazepine abuse, we studied how nicotine exposure in naive rats alters subsequent responses to the benzodiazepine diazepam, including diazepam-induced VTA neuron signaling and diazepam intake. Given that GABA_A_R signaling in VTA GABA neurons is impacted by chronic nicotine exposure (Thomas et al., 2018), we hypothesized that similar nicotine-induced neuroadaptations modify diazepam action on midbrain circuitry. Indeed, we found that a single, non-contingent nicotine exposure and volitional consumption of nicotine both dysregulate GABA_A_R signaling within VTA GABA neurons via downregulation of the K^+^, Cl^−^ co-transporter KCC2. This impaired Cl^−^ homeostasis and shifted diazepam-induced inhibition of VTA GABA neurons towards paradoxical excitation, which was associated with decreased DA neuron firing in response to diazepam. At the behavioral level, a single exposure to nicotine increased diazepam consumption, an effect that could be reversed by enhancing KCC2 function. These results indicate that nicotine exposure fundamentally alters the effects of diazepam on mesolimbic DA circuitry and thereby contributes to increased diazepam intake.

## Materials and Methods

### Animals

Male Long–Evans rats (Harlan-Envigo, weighing 300–500 g) were housed in a quiet, temperature-controlled and humidity-controlled facility under a 12/12 h light/dark cycle. Rats had food and water available *ad libitum* in their home cages. All rats were group housed except for use in behavior experiments when animals were transitioned to single-housing at the onset of daily drinking. All rats were handled at least 5 d prior to the beginning of testing. All animal procedures were performed in accordance with the University of Pennsylvania animal care committee’s regulations.

### Drugs and experimental design

Systemic administration of nicotine (0.4 mg/kg, freebase, i.p., Glentham Life Sciences) or saline (0.9% saline, i.p., Hospira) occurred 7–15 h prior to diazepam exposure or testing. For systemic administration, CLP290 was first dissolved in 40% β-cyclodextrin (20 mg/ml), then diluted in saline to a final concentration of 10 mg/ml in 20% β-cyclodextrin (Gagnon et al., 2013). Using 10 N NaOH, the pH was adjusted to be between 5 and 6. Systemic administration of CLP290 (10 mg/kg, i.p.) or vehicle (20% β-cyclodextrin) occurred 45 min prior to diazepam intake sessions over three non-consecutive days (Thomas et al., 2018). In *ex vivo* experiments, slices were incubated for 1 h in 10 μM CLP290, which was first dissolved in DMSO (100 mM), then diluted in artificial CSF (ACSF) to a final concentration. The carbonic anhydrase inhibitor acetazolamide (ACTZ) was bath applied at concentration of 10 μM. Diazepam (Sigma Aldrich) was dissolved in 190 proof ethanol before it was dissolved daily in the saccharin drinking solution. The final concentration of ethanol in the solution was 0.0475%. Drugs used for electrophysiological recordings were obtained from Sigma Aldrich unless otherwise specified. CLP290 was a generous gift from Dr. Y. De Koninck and Dr. A. Castonguay (Laval University, Quebec, Canada).

### *Ex vivo* electrophysiology

Horizontal slices (230 μm) containing the VTA were cut (Leica Microsystems) from adult and juvenile (P21–P28) Long–Evans rats in ice-cold, oxygenated (95% O_2_, 5% CO_2_), high-sucrose ACSF: 205.0 mM sucrose, 2.5 mM KCl, 21.4 mM NaHCO_3_, 1.2 mM NaH_2_PO_4_, 0.5 mM CaCl_2_, 7.5 mM MgCl_2_, and 11.1 mM dextrose. Immediately after cutting, slices were transferred to normal ACSF buffer: 120.0 mM NaCl, 3.3 mM KCl, 25.0 mM NaHCO_3_, 1.2 mM NaH_2_PO_4_, 2.0 mM CaCl_2_, 1.0 mM MgCl_2_, 10.0 mM dextrose, and 20.0 mM sucrose. The slices were constantly oxygenated (95% O_2_, 5%CO_2_) and maintained at 32°C in ACSF for 40 min, then at room temperature for at least 60 min. For incubation experiments, slices were bathed in CLP290 (10 μM) for an additional hour prior to recording. To perform electrophysiological recordings, slices were transferred to a holding chamber and perfused with normal ACSF at a constant rate of 2–3 ml/min at 32°C. Patch electrodes made of thin-walled borosilicate glass [1.12 mm inner diameter (ID), 1.5 mm outer diameter (OD); World Precision Instruments (WPI)] had resistances of 1.0–2.0 MΩ when filled with the internal solution: 135.0 mM KCl, 12.0 mM NaCl, 2.0 mM Mg-ATP, 0.5 mM EGTA, 10.0 mM HEPES, and 0.3 mM Tris-GTP (pH 7.2–7.3).

For E_GABA_ perforated-patch recordings in VTA GABA neurons, gramicidin was first dissolved in methanol to a concentration of 10 mg/ml and then diluted in a pipette solution to a final concentration of 150 μg/ml. For synaptic stimulation recordings, a bipolar tungsten-stimulating electrode (World Precision Instruments) was placed 100–150 μm away from the recording electrode. To determine E_GABA_, evoked IPSCs (eIPSCs) were measured under voltage clamp at different holding potentials. Amplitudes of eIPSCs were plotted against voltage to estimate the reversal potential. After each perforated-patch experiment, recordings were converted to the whole-cell configuration, and the hyperpolarization-activated current (I_h_) was measured. Recordings were performed in the presence of 6,7-dinitroquinoxaline-2,3-dione (DNQX; 20 μM) and DL-2-amino-5-phosphonopentanoic acid (AP5, 50 μM; Tocris Bioscience), CGP55845 (1 μM), and tetrodotoxin (0.5 μM, Abcam) to isolate GABAergic currents.

Analogous experiments were conducted to determine whether acute nicotine altered E_GABA_ in young rats (P21–P28). Indistinguishable from adult animals, VTA GABA neurons from P21 to P28 rats receiving nicotine showed a significantly more depolarized E_GABA_ value compared with saline-treated controls: −63.8 ± 4.3 mV after nicotine, −87.6 ± 2.4 mV after saline, *n* = 7, *n* = 8 cells/group, *n* = 4, *n* = 5 rats/group, *t* = 4.956, *p* = 0.000 (Table 1, line a). Cell visualization and long-term patch-clamp recordings in slices from adult rats are harder compared with juvenile rats. Therefore, given the similarity of nicotine effects between young and adult rats, subsequent *ex vivo* electrophysiological recordings were performed in midbrain slices from juvenile rats.

**Table 1.** Statistical table

	Data structure	Type of test	*p* value	Effect size	Power
a	Normal	*t* test	0.000	3.438	1.000
b	Normal	*t* test	7.039 × 10^–8^	3.362	1.000
c	Normal	*t* test	0.245	0.582	0.307
d	Normal	ANOVA repeated measures	0.736	0.022	0.164
e	Normal	ANOVA repeated measures	0.000	0.357	1.000
f	Normal	Paired *t* test	0.006	1.848	0.946
g	Normal	Paired *t* test	0.033	1.196	0.652
h	Normal	*t* test	0.000	3.430	1.000
i	Normal	Paired *t* test	0.047	1.069	0.559
j	Normal	Paired *t* test	0.049	1.056	0.549
k	Normal	*t* test	0.137	0.816	0.440
l	Normal	ANOVA repeated measures	0.000	0.449	1.000
m	Normal	*t* test	0.001	1.232	0.936
n	Normal	*t* test	0.817	0.085	0.056
o	Normal	*t* test	0.965	0.016	0.050
p	Normal	*t* test	0.000	3.869	1.000
q	Normal	*t* test	0.674	0.123	0.062
r	Normal	*t* test	0.491	0.379	0.100
s	Normal	*t* test	0.232	0.826	0.315
t	Normal	*t* test	0.926	0.133	0.056
u	Normal	*t* test	0.001	2.202	0.965
v	Normal	ANOVA repeated measures	0.000	0.265	1.000
w	Normal	ANOVA repeated measures	0.029	0.252	0.616
x	Normal	*t* test	0.029	1.617	0.912
y	Normal	ANOVA repeated measures	0.940	0.000	0.051
z	Normal	*t* test	0.940	0.034	0.051
aa	Normal	ANOVA repeated measures	0.040	0.165	0.551
bb	Normal	*t* test	0.040	0.802	0.491

Effect sizes and power were calculated with G*Power 3.1.

To measure activity-dependent depression of eIPSCs, whole-cell recordings were performed during repetitive stimulation. The estimated GABA reversal was approximately −70 mV, and the internal solution contained the following: 123.0 mM K^+^-gluconate, 8.0 mM NaCl, 2.0 mM Mg-ATP, 0.2 mM EGTA, 10.0 mM HEPES, and 0.3 mM Tris-GTP (pH 7.2–7.3). Synaptic GABA_A_ input was isolated using DNQX, AP5, and CGP55845. The liquid junction potential between the bath and the pipette solutions was corrected.

The effects of diazepam on firing rates of VTA GABA neurons were recorded in cell-attached configuration in passive voltage-follower mode. For focal puff application of diazepam, the puff capillary was placed 10–20 μm from the patched neuron. Brief (150–300 ms) focal pressure ejections (one to three puffs at 1.3 Hz) of diazepam (0.5 mM) occurred at 1 min intervals following recording of baseline firing. After each cell-attached experiment, recordings were converted to the whole-cell configuration and I_h_ was measured. Some cells were also backfilled with neurobiotin for immuno-identification.

Spontaneous IPSCs (sIPSCs) onto VTA DA neurons were recorded in voltage-clamp mode in whole-cell configuration. Synaptic GABA_A_ inputs were isolated pharmacologically with the AMPA and NMDA-type glutamate receptor antagonists DNQX and AP5. Additional sIPSC experiments were also performed with ACTZ (10 μM) in the bath or CLP incubation (10 μM).

**Figure 1.**
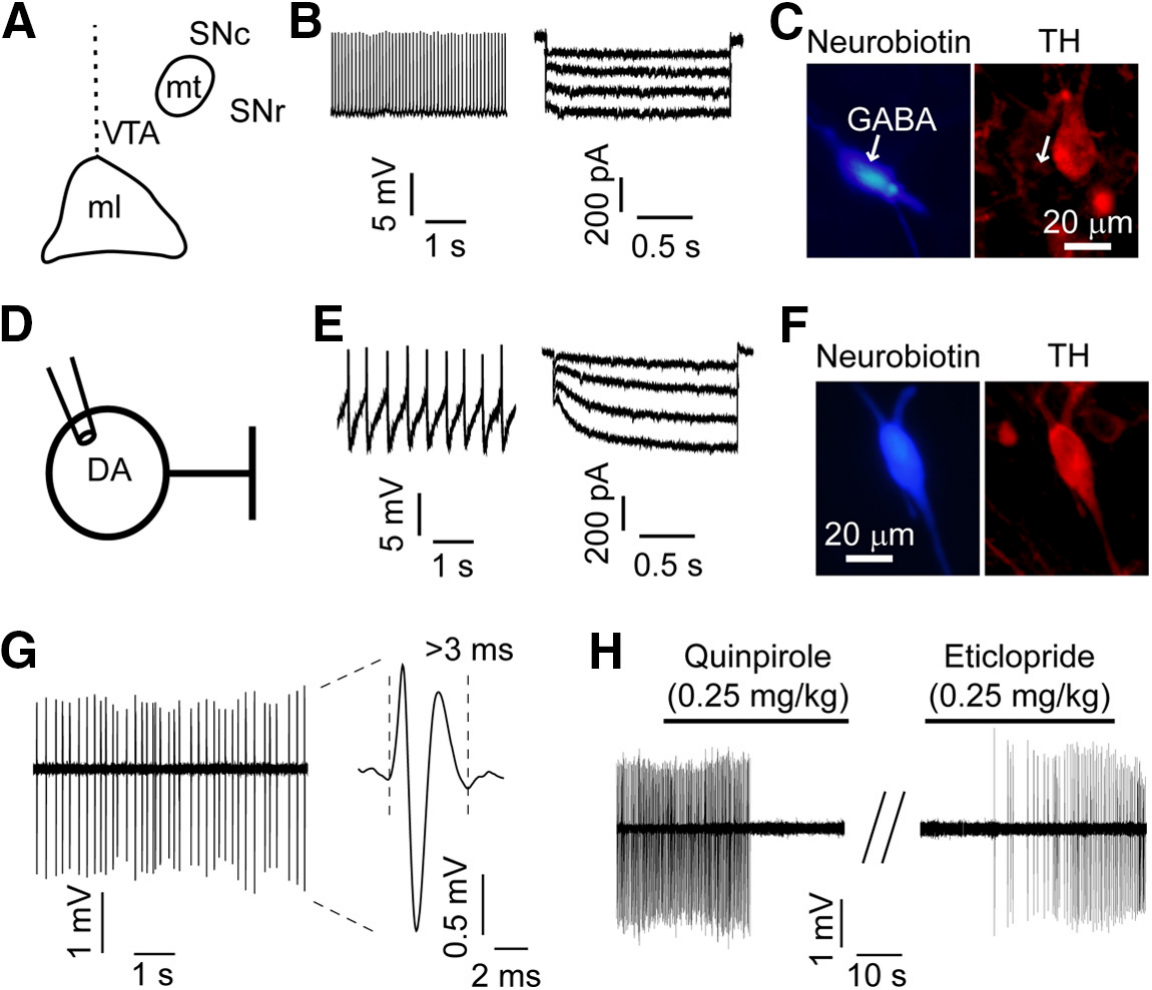
*Ex vivo* and *in vivo* identification of GABA and DA neurons in the lateral VTA. ***A***, The lateral VTA was identified as medial to the medial terminal nucleus of the accessory optic tract (mt) and lateral and rostral to the crest of medial lemniscus (ml). SNc = substantia nigra pars compacta; SNr = substantia nigra pars reticulata; ml = medial lemniscus; mt = medial terminal nucleus of accessory optic tract. ***B***, VTA GABA neurons displayed fast spontaneous firing rates (>7 Hz) and lack of I_h_. The firing rates of VTA GABA neurons were recorded in cell-attached configuration in passive voltage-follower mode. I_h_ was recorded in whole-cell configuration using voltage step protocol (−40 to −110 mV in 10-mV steps, 1.5-s duration, traces on ***B*** displayed for steps from −80 to −110). ***C***, Neurobiotin-labeled VTA neurons with these electrophysiological properties were immunonegative for TH, consistent with a non-DA phenotype. ***D***, *Ex vivo* patch clamp recordings of DA neurons were performed in the lateral VTA. Spontaneous firing rate of DA neurons was measured using the cell-attached configuration, and the whole-cell configuration was used to identify DA neurons electrophysiologically and histochemically on termination of the recording. ***E***, VTA DA neurons displayed low spontaneous firing frequency (<5 HZ) and large I_h_ (>150 pA). ***F***, Neurobiotin-labeled DA neurons with these electrophysiological properties were immuno-positive for TH (red stain). ***G***, *In vivo* single-unit recordings of VTA DA neurons in anesthetized rats. Example traces for putative DA neurons showing low-frequency spontaneous firing (<10 Hz) and broad triphasic action potential (>3 ms). ***H***, Putative DA neurons were inhibited by the D2-type receptor agonist quinpirole (0.25 g/kg). This effect was then reversed following the D2-type receptor antagonist eticlopride (0.25 g/kg).

All electrophysiological recordings were performed in the lateral VTA (Fig. 1*A*). VTA GABA neurons were identified by a combination of factors, including small somata size, high firing rate (>7 Hz), and the lack of I_h_ (Fig. 1*B*). Cells with these properties were consistently tyrosine hydroxylase (TH) negative (>95%, Fig. 1*C*; Klink et al., 2001; Korotkova et al., 2006; Margolis et al., 2006; Ostroumov et al., 2016). Alternatively, DA neurons were identified in the lateral VTA by their morphology (>20-μm soma size), their low firing frequency (<5 Hz), and the presence of a large I_h_ (Fig. 1*D*,*E*). Together, these criteria are shown to correlate with TH-positive cells using immunocytochemistry approaches (95%; Fig. 1*F*; Chen et al., 2008; Zhang et al., 2010; Ostroumov et al., 2016).

Recordings were made using an Axopatch 200B amplifier (Molecular Devices), filtered at 10 kHz, digitized at 20 kHz using pClamp 9.2 (Digidata Interface, Molecular Devices), and analyzed off-line using Clampfit 9.2.

### Western blotting

The VTA was harvested in horizontal brain slices from adult rats and prepared as described previously (see above, *Ex vivo* electrophysiology). Membrane fractions were prepared using the Mem-PER Plus Membrane Protein Extraction kit (model 89 842; Thermo Scientific). Samples (30-μg protein) in 2.5% 2-mercaptoethanol were run through a 4%–15% Precast Protein Gel (#4561083; Bio-Rad) and transferred to nitrocellulose membrane (Bio-Rad). The primary antibodies used were rabbit anti-KCC2 antibody at 1:400 (#07-432; Millipore) and mouse anti-glyceraldehyde 3-phosphate dehydrogenase (GAPDH) antibody (#MAB374; Millipore) at 1:400. The secondary antibodies used were goat anti-rabbit immunoglobulin (Ig)G secondary antibody (#T2191; Applied Biosystems) or goat anti-mouse IgG/IgM (#T2192, Applied Biosystems). All antibodies were diluted in SignalBoost solution (#407207; EMD Millipore). Membranes were developed using Tropix CDP-Star solution (T2218; Applied Biosystems) for 5 min and then scanned using the Protein Simple FluorChem R chemiluminescence detector and analyzed using ImageJ. The optical densities of KCC2-specific bands were measured and normalized to the loading control GAPDH values.

### *In vivo* electrophysiology

Rats were anesthetized with isoflurane and implanted with a catheter in the jugular vein. Animals were positioned on a stereotaxic apparatus and an incision in the middle was made to expose the skull. Burr holes were drilled to accommodate recording and ground electrodes. Rat body temperature was maintained throughout the experiment at 37ºC using an isothermal pad (Braintree Scientific).

Electrodes were pulled on a horizontal puller (Sutter Instrument) from filament-containing borosilicate glass (0.68 mm ID, 1.2 mm OD; WPI). Electrodes were backfilled with 0.5 M Na^+^ acetate and 2% Chicago sky blue (5–15 MΩ). Electrodes were slowly lowered with a micromanipulator and positioned in the lateral VTA (coordinates 5.3–6.0 mm posterior from bregma, 0.8–1.4 mm lateral to midline and 7.5–8.5 mm ventral to brain surface). Electrical signals were recorded using an AM Systems Model 1700 amplifier, filtered at 0.3–5 kHz and monitored using pClamp 8.0 (Molecular Devices) and an audiomonitor (Grass Instruments).

Lateral VTA DA neurons were identified based on their electrophysiological and pharmacological properties. Putative DA neurons exhibited low-frequency spontaneous firing (<10 Hz, irregular or bursting) and a broad action potential waveform (>3 ms; Fig. 1*G*). In addition to electrophysiological criteria, we used pharmacological methods to identify DA neurons. DA neurons included in this study decreased their firing in response to D2 receptor agonist (quinpirole, 0.25 mg/kg, i.v.), and this effect was reversed by application of D2 receptor antagonist (eticlopride, 0.25 mg/kg, i.v.; Fig. 1*H*). This combination of conservative electrophysiological and pharmacological criteria is a reliable methodology to identify DA neurons in the lateral VTA (Ungless and Grace, 2012).

After 6–20 min of stable neuronal recording (basal activity), a 0.25 mg/kg dose of diazepam was injected intravenously. Only one cell was recorded per rat. Drug-induced modifications of the basal activity were calculated in percentage for the 3- to 7-min period following each administration of diazepam and compared with the predrug baseline. Following diazepam administration, quinpirole and eticlopride were injected intravenously for pharmacological identification of VTA neurons.

After recording, the cell location was labeled by application of Chicago sky blue dye. The dye was injected via administering positive pressure (1–2 min) through the suction port in the microelectrode holder. At the end of experiment, brains were kept in 10% formalin for at least 1 d. The brains were cut into 75-μm coronal sections on a vibratome (Leica Microsystems Inc.) and stained with cresyl violet to verify the recording site by light microscopy.

### Limited access nicotine consumption

Initially, rats received 1-h access to saccharin solution (0.125%) in a chamber distinct from their home cage. Baseline saccharin consumption was monitored for at least 5 d until intake appeared stable. Nicotine was then introduced into the saccharin drinking solution in the following way: 0.004 mg/ml nicotine (freebase) on day 1 and 0.008 mg/ml nicotine (freebase) for the subsequent 6 d. The control group was never introduced to nicotine and received saccharin control solution for the same number of sessions. Similar to the baseline days, rats also had 1-h access to these nicotine or saccharin solutions for the 7 d. Western blotting or electrophysiological measurements were performed the day after the last nicotine or saccharin session.

### Limited access diazepam consumption

All rats were handled and habituated to injections for 5 d prior to the start of experiments. Each day at the onset of the dark cycle (7 P.M.), water bottles were replaced with bottles containing 0.125% saccharin dissolved in water. Solution intake was measured 2 h later by subtracting the weight of the bottles at 9 P.M. from the initial weight of the bottles before placement in the cages at 7 P.M. An empty control cage was used to account for any leakage from the experimental bottles. Saccharin drinking proceeded for 7–10 d until the stable baseline drinking levels were achieved (defined as ±1 SD of the last three saccharin days for each rat).

The day following the final baseline session of saccharin drinking, rats were injected with either nicotine or saline, intraperitoneally, 7 h prior to drinking. Diazepam was then introduced into the drinking solution that night at 7 P.M. Diazepam was dissolved to a concentration of 0.005 mg/ml in the saccharin drinking solution. Doses of diazepam achieved were expressed as mg/kg. Rats whose baseline saccharin drinking prior to diazepam exposure fell outside of our acceptable range of saccharin consumption, defined as 2 SD above and below the mean baseline saccharin consumption of all rats (14.1 g), did not receive diazepam or proceed with the experiment. This was done in order to ensure that rats were consuming enough solution to achieve a pharmacologically relevant dose of diazepam.

### Statistical analyses

A two-tailed *t* test was used to assess differences between the mean GABA reversal potential, sIPSC frequency, and DA neuron firing rate. For Western blot analysis, a paired *t* test was used to compare protein levels from saline-treated and nicotine-treated littermates that were run on the same gel. An ANOVA with repeated measures was used to analyze the repetitive synaptic stimulation experiment, action potential firing in VTA GABA neurons, and daily diazepam intake. For analysis of action potential firing, the raw data were converted into percent of basal, and the last three bins (2 min each) before bath application of diazepam were used as the baseline. Significance for all analyses was determined by *p* < 0.05. All statistical analysis was performed using SPSS software (IBM Corp.).

## Results

### Nicotine impairs Cl^−^ homeostasis in VTA GABA neurons

**Figure 2.**
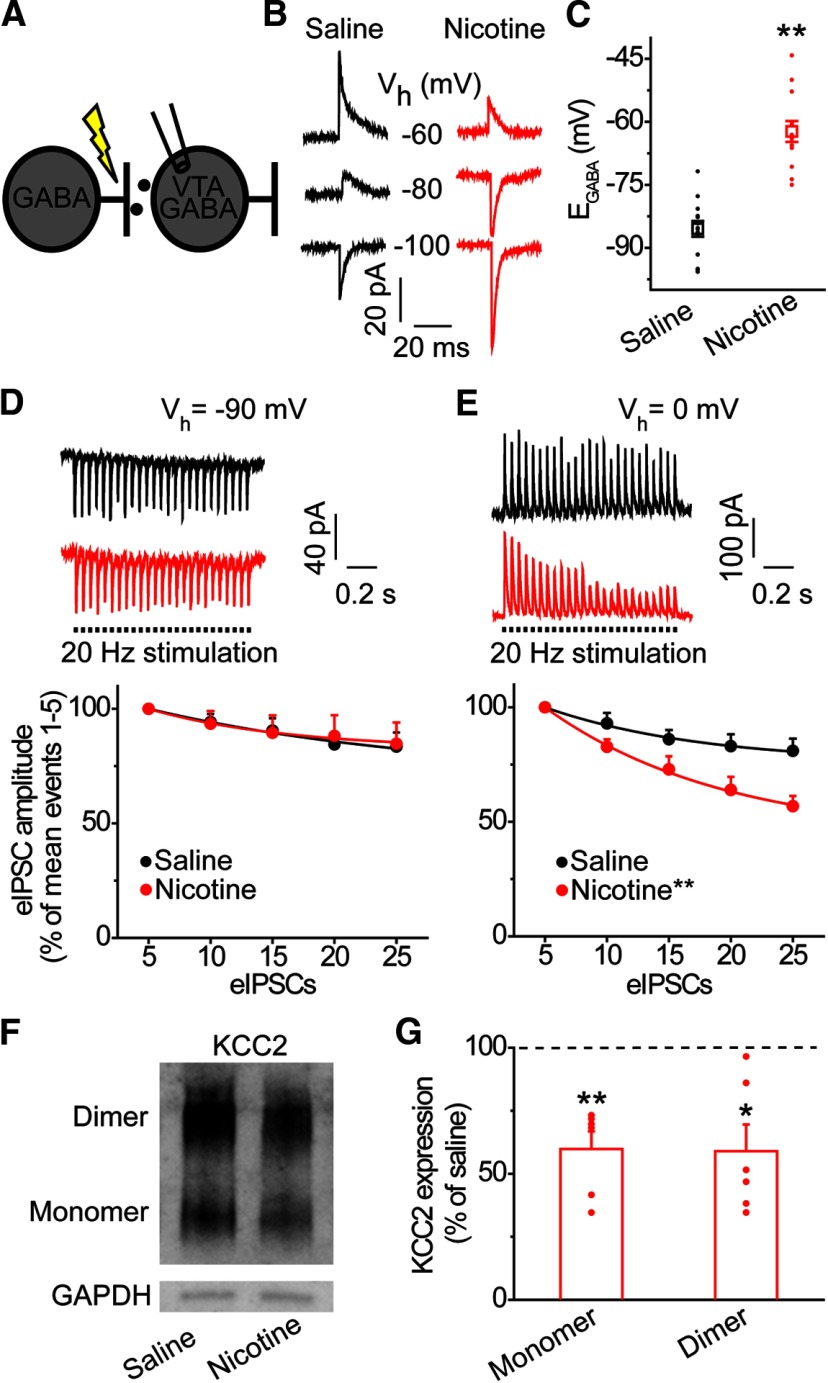
Acute nicotine exposure alters Cl^−^ homeostasis in the VTA. ***A***, GABAergic input onto VTA GABA neurons was measured using gramicidin perforated-patch whole-cell recordings at different holding potentials to measure nicotine-induced alterations in anion homeostasis. GABA_A_ IPSCs were evoked by electrical stimulation in the presence of ionotropic glutamate and GABA_B_ receptor antagonists. ***B***, Representative eIPSC recordings from saline (black) and nicotine (red)-treated animals at the given holding potentials (V_h_). The eIPSCs reverse direction at E_GABA_. For display, the traces were filtered, and stimulus artifacts were removed. ***C***, VTA GABA neurons from nicotine-treated animals (red) showed a significantly more positive E_GABA_ compared with saline-treated animals (black); **p < 0.01, significantly different by *t* test, *n* = 13, *n* = 14 cells/group, *n* = 7 rats/group. ***D***, Activity-dependent synaptic depression in VTA GABA neurons was measured during whole-cell patch clamp recordings under repetitive GABA_A_R stimulation. Traces on top: representative GABA neurons from saline (black) and nicotine-treated rats (red) demonstrated a similar depression in eIPSC amplitude when stimulated at 20 Hz and clamped at −90 mV. Bottom graph, At −90 mV, VTA GABA neurons from control and stressed animals showed no significant difference in rate of eIPSC amplitude depression (*p* > 0.05, *n* = 8 cells, *n* = 4 rats for the nicotine group and *n* = 16 cells, *n* = 6 rats for the saline group). Amplitude values were averaged for five eIPSCs and shown as a percent of the first five eIPSCs mean amplitude. ***E***, Cl^−^ accumulation was measured as in ***D***, but VTA GABA neurons were clamped at 0 mV. Top traces, Upon stimulation, VTA GABA neuron from control animal (black) demonstrated a minor depression of eIPSC amplitude compared with the significantly greater depression seen in a GABA neuron from a stressed animal (red). Bottom graph, At 0 mV, GABA neurons from nicotine treated animals (red) demonstrated a significantly greater rate of eIPSC amplitude depression than GABA neurons from saline-treated animals (black); **p < 0.01, significantly different by ANOVA with repeated measures, *n* = 8 cells, *n* = 4 rats for the nicotine group and *n* = 16 cells, *n* = 6 rats for the saline group. ***F***, Western blot analysis was conducted for KCC2 protein expression with GAPDH as a loading control. A representative Western blotting shows reduced expression of KCC2 in nicotine-treated animals. ***G***, Densiometric analysis showed a significant reduction in KCC2 protein in nicotine-treated animals (red bars) compared with saline-treated controls (horizontal dashed line); *p < 0.05, **p < 0.01, significantly different by paired *t* test, *n* = 6 animals/group.

A recent study showed that repeated nicotine exposure during adolescence modifies inhibitory synaptic transmission in the VTA via depolarizing shifts in the GABA_A_R reversal potential (E_GABA_) of GABA neurons (Thomas et al., 2018). To determine whether acute nicotine exposure was sufficient to alter E_GABA_, rats were treated with nicotine (0.4 mg/kg) or saline ∼15 h prior to electrophysiological recordings from midbrain slices. We chose the 15-h pretreatment period to examine the lasting impact of nicotine on neural circuits, and not the immediate pharmacological effect of nicotine itself. We measured GABA_A_R currents at different membrane potentials following electrical stimulation (Fig. 2*A*,*B*). VTA GABA neurons from rats receiving nicotine showed a significantly more depolarized E_GABA_ value compared with saline-treated controls (Fig. 2*B*,*C*): −62.3 ± 2.5 mV after nicotine (red data) versus −85.5 ±1.9 mV after saline (black data), *n* = 13, *n* = 14 cells/group, *n* = 7 rats/group, *t* = 7.525, *p* = 7.039 × 10^−8^ (Table 1, line b). Although E_GABA_ was depolarized, acute nicotine did not alter the membrane resting potential (measured as zero holding current): −62.7 ± 2.7 mV after nicotine, −58.8 ± 1.8 mV after saline, *n* = 13, *n* = 14 cells/group, *n* = 7 rats/group, *t* = −1.191, *p* = 0.245 (Table 1, line c).

A depolarizing shift in E_GABA_ reflects a higher intracellular anion concentration and is often mediated by a decrease in Cl^−^ extrusion capacity. During prolonged GABA_A_R stimulation, decreased Cl^−^ extrusion capacity leads to intracellular Cl^−^ accumulation, culminating in the collapse of the Cl^−^ gradient and decreased synaptic GABA_A_R inhibition. To test whether acute nicotine administration weakens Cl^−^ extrusion in VTA GABA neurons, we established a similar Cl^−^ gradient in cells from nicotine and saline-treated rats by using the whole-cell patch clamp recording configuration. Then, we applied repetitive GABA_A_R stimulation to measure activity-dependent collapse of the Cl^−^ gradient, which is reflected in the depression of the eIPSCs (Hewitt et al., 2009; Ostroumov et al., 2016; Ferrini et al., 2017). Specifically, the rate of decrease of eIPSC amplitude at the conditions that favor Cl^−^ influx (0 mV) depends on intracellular Cl^−^ accumulation and activity-dependent synaptic depression. In contrast, the rate of eIPSC amplitude decrease at the conditions of Cl^−^ efflux (−90 mV) only reflect activity-dependent synaptic depression. Upon electrical stimulation at 20 Hz at a holding potential of −90 mV, nicotine did not affect the rate of synaptic depression in VTA GABA neurons (Fig. 2*D*): group × number, *F*_(4,88)_ = 0.499, *p* = 0.736 (Table 1, line d). In contrast, the decrease in eIPSC amplitude at 0 mV occurred significantly faster after nicotine pretreatment (Fig. 2*E*): group × number, *F*_(4,88)_ = 12.240, *p* = 0.000 (Table 1, line e). The differential effect of acute nicotine at −90 versus 0 mV indicates that nicotine increased postsynaptic Cl^−^ accumulation, suggesting a reduced capacity for Cl^−^ extrusion in VTA GABA neurons.

Reductions in Cl^−^ extrusion capacity in neurons are often mediated by the downregulation of KCC2 (Ostroumov et al., 2016; Thomas et al., 2018). Within the VTA, KCC2 protein is expressed on non-DA, GABAergic neurons, which is consistent with the presence of another chloride extrusion mechanism in DA neurons (Gulácsi et al., 2003; Ostroumov et al., 2016; Taylor et al., 2016; Thomas et al., 2018). To examine acute nicotine-induced alterations in KCC2 protein expression, we performed Western blot analysis using an antibody against KCC2 protein. Immunoblots revealed two prominent bands (∼140 and ∼270 kDa), indicating the presence of monomeric and dimeric structures of KCC2 protein (Fig. 2*F*). A significant reduction in the expression of KCC2 was observed after acute nicotine pretreatment (Fig. 2*G*): 59.9 ± 7.0% for monomer, 59.0 ± 10.6% for dimer, *n* = 6 rats/group, *t* = 4.526, *p* = 0.006 for monomer (Table 1, line f), *t* = 2.930, *p* = 0.033 for dimer (Table 1, line g). Our results indicate that acute nicotine decreases KCC2 expression and function, leading to impaired Cl^−^ homeostasis in VTA GABA neurons.

**Figure 3.**
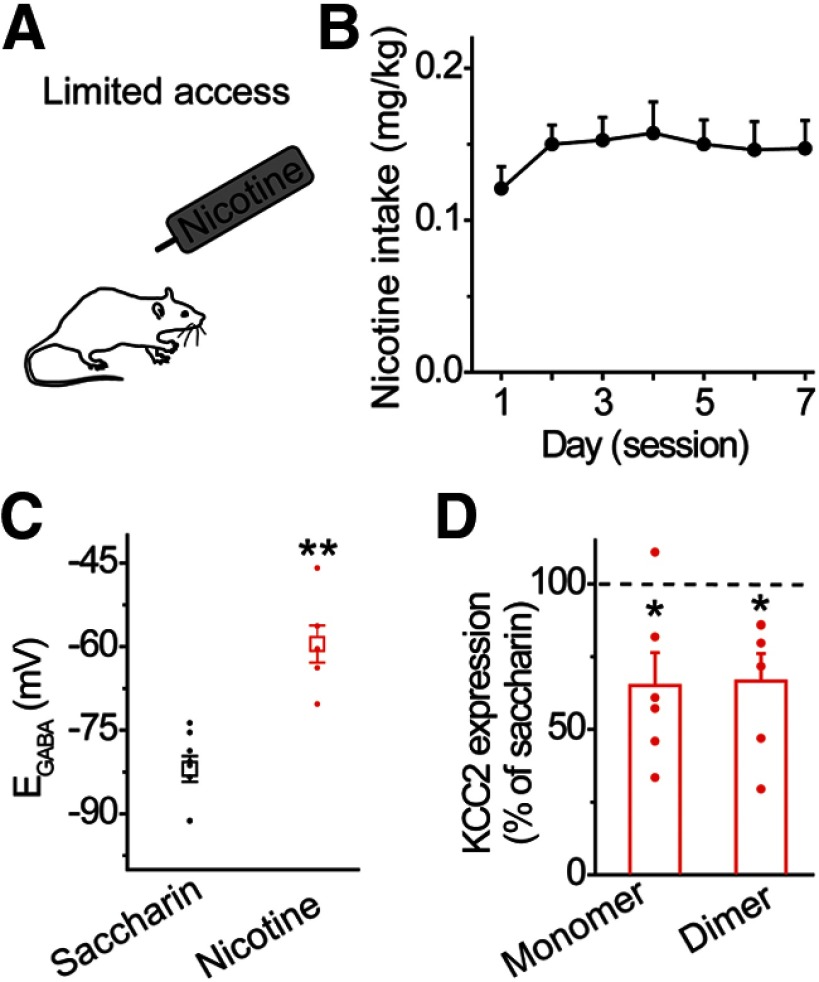
Nicotine consumption impairs chloride homeostasis in the VTA. ***A***, Rats consumed a nicotine-containing saccharin solution for 1 h/d over the course of 7 d. ***B***, Mean nicotine consumption during 1 h daily free-access sessions. Rats consumed 0.15 ± 0.01 mg/kg nicotine on average over the 7 d of limited access, *n* = 11 rats. ***C***, VTA GABA neurons from nicotine-drinking (red) rats displayed a significantly depolarized E_GABA_ relative to saccharin-drinking (black) rats; **p < 0.01, significantly different by *t* test, *n* = 6, *n* = 8 cells/group, *n* = 3 rats/group. ***D***, Western blot analysis revealed that nicotine-drinking produced a significant reduction in KCC2 expression in the VTA relative to saccharin drinking rats (horizontal dashed line); *p < 0.05 significantly different by paired *t* test, *n* = 6 animals/group.

In addition to acute noncontingent nicotine exposure (i.e., experimenter-administered), we measured chloride homeostasis in rats that received 1-h daily access to nicotine in saccharin drinking solution. Under this paradigm, rats demonstrated stable intake of nicotine (0.15 ±0.01 mg/kg, *n* = 11 rats; Fig. 3*A*,*B*). Seven nicotine drinking sessions led to a significantly depolarized E_GABA_ value in VTA GABA neurons compared with seven drinking sessions of saccharin alone (Fig. 3*C*): −59.5 ± 3.3 mV after nicotine (red data) versus −82.0 ± 2.3 mV in saccharin controls (black data), *n* = 6, *n* = 8 cells/group, *n* = 3 rats/group, *t* = 5.724, *p* = 9.544 × 10–5 (Table 1, line h). In addition, we observed a significant reduction in KCC2 expression within the VTA after 7 d of nicotine consumption (Fig. 3*D*): 65.0 ± 11.3% for monomer, *t* = 2.618, *p* = 0.047; 66.6 ± 9.5% for dimer, *t* = 2.587, *p* = 0.049, *n* = 6 rats/group (Table 1, lines i, j). Taken together, these data indicate that different methods of nicotine administration induce similar alterations in KCC2 expression, chloride homeostasis, and GABA_A_R signaling within VTA GABA neurons.

### Acute nicotine exposure shifts the effect of diazepam in VTA GABA neurons

**Figure 4.**
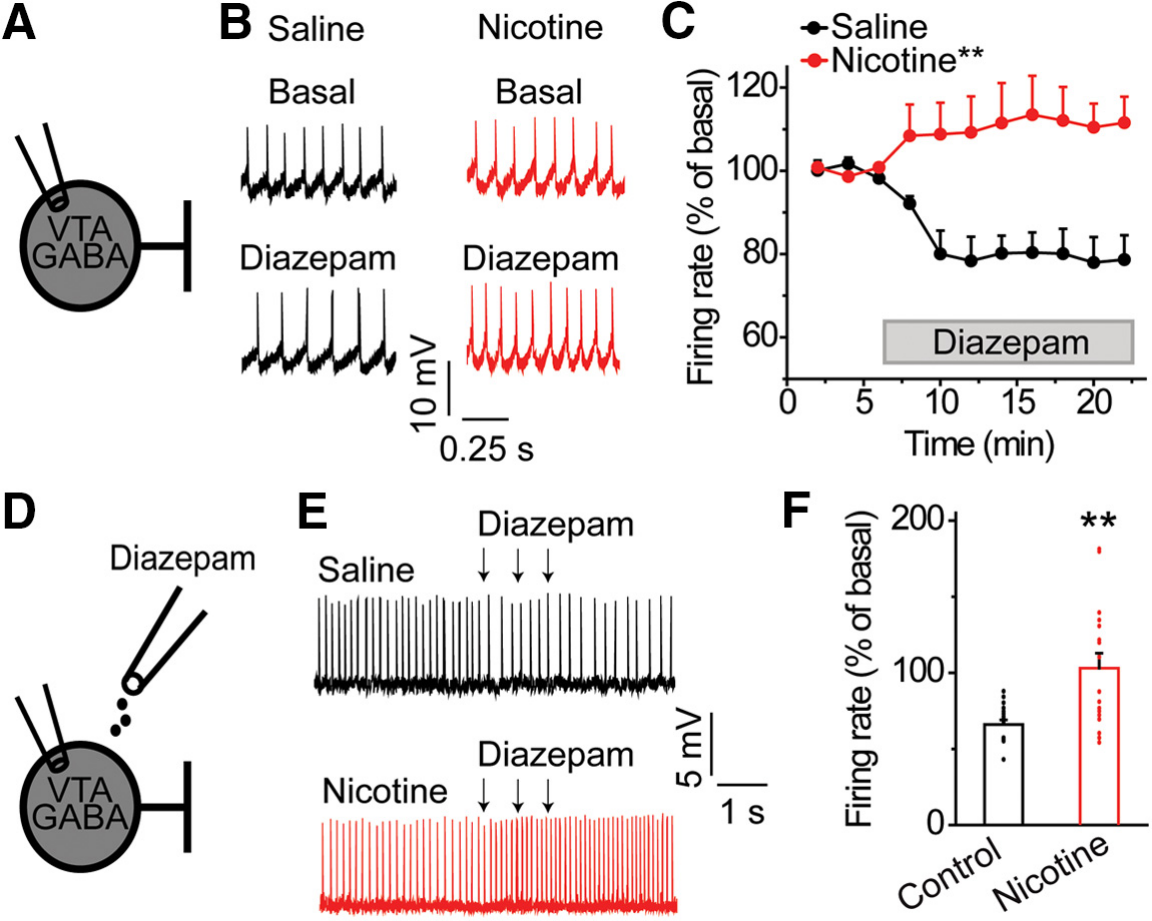
Diazepam increases the firing rate of VTA GABA neurons following acute nicotine exposure. ***A***, Spontaneous firing rates of VTA GABA neurons were measured using the cell-attached configuration before and after bath application of diazepam. ***B***, Representative recordings from VTA GABA neurons before and after bath administration of diazepam in the nicotine (red) and saline (black) groups. After nicotine pretreatment, bath applied diazepam increased GABA neuron firing rates in contrast to decreased firing in the saline group. ***C***, In saline-treated controls, diazepam decreased the firing rate of VTA GABA neurons. In the nicotine-treated group, diazepam increased the firing rate of VTA GABA neurons; **p < 0.01, significantly different by ANOVA with repeated measures, *n* = 7, *n* = 8 cells/group, *n* = 4, *n* = 5 rats/group. ***D***, Cell-attached recordings were performed on VTA GABA neurons to assess changes in firing in response to locally applied diazepam *ex vivo*. ***E***, VTA GABA neurons from saline-treated animals (black) demonstrated a reduction in action potential firing when diazepam was focally puffed onto the recorded neuron. In contrast, VTA GABA neurons from nicotine-treated rats (red) often displayed enhanced firing to diazepam puff application. ***F***, Focal puff application of diazepam decreased the firing rate of VTA GABA neurons in saline-treated controls. In nicotine-treated animals, puff-administered diazepam increased the firing rate of nearly half of all VTA GABA neurons recorded; **p < 0.01, significantly different by *t* test, *n* = 17 cells/group, *n* = 9, *n* = 11 rats/group.

Depolarizing shifts in E_GABA_ result in decreased synaptic inhibition or even paradoxical GABAergic excitation of VTA GABA neurons in response to GABA_A_R activation (Ostroumov et al., 2016; Thomas et al., 2018). Given that benzodiazepines enhance GABA_A_R function in VTA GABA neurons (Tan et al., 2010), we hypothesized that acute nicotine exposure would alter the effects of diazepam on VTA GABA neuron activity. To test this, midbrain slices were prepared from rats pretreated with nicotine or saline (15 h prior), and diazepam’s effect on VTA GABA neuron spontaneous firing rate was measured in a cell-attached configuration (Fig. 4*A*). The basal spontaneous firing rate of GABA neurons was not altered after exposure to the nicotine treatment: 12.9 ± 1.3 Hz in saline-treated versus 10.2 ± 1.2 Hz in nicotine-treated rats (*n* = 7, *n* = 8 cells/group, *n* = 4, *n* = 5 rats/group, *t* = −1.584, *p* = 0.137; Table 1, line k). Bath-application of diazepam (5 μM) on VTA slices from saline-treated control animals decreased GABA neuron firing rate (Fig. 4*B*,*C*, black data), consistent with the enhanced GABA_A_R-mediated inhibition of VTA GABA neurons (Tan et al., 2010). In marked contrast, a significant diazepam-induced increase in GABA neuron firing rate was observed following nicotine (Fig. 4*B*,*C*, red trace): group × time, *F*_(10,130)_ = 10.593, *p* = 0.000 (Table 1, line l). This finding demonstrates that diazepam-dependent enhancement of GABA_A_R activity induced GABAergic excitation of VTA GABA neurons after exposure to nicotine.

To differentiate global diazepam-mediated alterations in VTA GABA neuron firing from local synaptic effects on the patched neurons, we performed brief focal pressure ejection of diazepam onto VTA GABA neurons (Fig. 4*D*). Upon focal application of diazepam, VTA GABA neurons from control animals showed decreased firing (Fig. 4*E*,*F*; black data, 66.0 ± 3.0% of basal firing, *n* = 17 cells, *n* = 11 rats). After acute nicotine treatment, local diazepam application produced less GABA-neuron inhibition, and eight out of 17 GABA neurons from nicotine-treated animals showed increased firing to diazepam (Fig. 4*E*,*F*, red data; 103 ± 9.9% of basal, *n* = 17 cells, *n* = 9 rats, *t* = 3.592, *p* = 0.001; Table 1, line m). Taken together, our results show qualitatively similar effects of bath and local diazepam application on VTA GABA neuron firing rate.

### Acute nicotine increases diazepam-induced inhibition of DA neurons *ex vivo*

**Figure 5.**
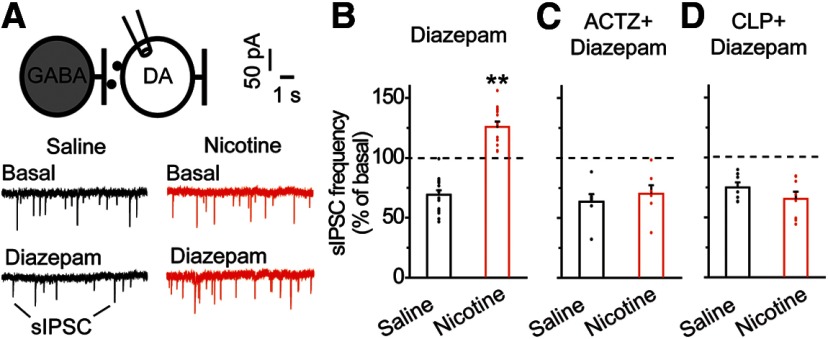
Acute nicotine exposure increases diazepam-induced GABA transmission onto VTA DA neurons. ***A***, sIPSCs onto VTA DA neurons were recorded using the whole-cell patch clamp configuration. Shown are representative recordings of sIPSCs before and after diazepam administration in the nicotine-treated (red) and saline (black) groups. ***B***, Mean changes in sIPSC frequency after bath application of diazepam in VTA DA neurons. DA neurons from saline-treated (black) rats displayed a reduction in sIPSC frequency on diazepam application. In contrast, DA neurons from nicotine-treated (red) animals showed a significantly increased diazepam-induced sIPSC frequency; **p < 0.01, significantly different by *t* test, *n* = 14, *n* = 15 cells/group, *n* = 6 rats/group. ***C***, Following bath application of ACTZ, diazepam-induced sIPSC frequency in VTA DA neurons did not differ between saline-treated (black) and nicotine-treated (red) groups (*p* > 0.05, *n* = 7 cells/group, *n* = 3 rats/group). ***D***, Following CLP290 incubation, no significant difference in diazepam-induced sIPSC frequency was noted among DA neurons from the saline (black) or nicotine (red) groups (*p* > 0.05, *n* = 7, *n* = 8 cells/group, *n* = 3, *n* = 4 rats/group).

VTA GABA transmission influences midbrain DA neuron activity and is modulated on exposure to diazepam (Tan et al., 2010). Depolarizing shifts in E_GABA_ within VTA GABA neurons were previously associated with greater ethanol-induced inhibition of lateral VTA DA neurons (Ostroumov et al., 2016; Thomas et al., 2018). To determine whether acute nicotine exposure altered diazepam-induced GABA release onto DA neurons, we performed whole-cell patch-clamp recordings of lateral VTA DA neurons and measured sIPSCs (Fig. 5*A*). No significant differences in baseline sIPSC frequency or amplitude were detected between the saline and nicotine groups: 2.2 ± 0.2 Hz in saline-treated versus 2.3 ± 0.4 Hz in nicotine-treated rats, 34.4 ± 4.0 pA in saline-treated versus 34.2 ± 2.8 pA in nicotine-treated rats (*t* = 0.234, *p* = 0.817 for frequency and *t* = −0.045, *p* = 0.965 for amplitude, *n* = 14, *n* = 15 cells, *n* = 6 rats/group, Table 1, lines n, o). In saline-treated control rats, bath-applied diazepam (5 μM) produced a decrease in sIPSC frequency (Fig. 5*A*,*B*): 69.1 ± 3.8% of basal, black data, *n* = 15 cells, *n* = 6 rats. In contrast, lateral-VTA DA neurons from rats pretreated with nicotine showed a significant diazepam-induced increase in sIPSC frequency compared with control rats (Fig. 5*A*,*B*): nicotine-treated, 125.8 ± 4.4% of basal, red data, *n* = 14 cells, *n* = 6 rats, *t* = 9.850, *p* = 0.000 (Table 1, line p). Importantly, neither group showed alterations in sIPSC amplitude on exposure to diazepam: 103.36 ± 11.3% in saline-treated rats versus 98.0 ± 4.9% in nicotine-treated rats (*n* = 14, *n* = 15 cells/group, *n* = 6 rats/group, *t* = −0.425, *p* = 0.674; Table 1, line q). An increase in the frequency, but not the amplitude, of sIPSCs following diazepam suggests enhanced presynaptic GABA release onto the DA neurons. These changes in GABA release in saline-treated and nicotine-treated groups correspond with diazepam-induced inhibition and excitation of VTA GABA neurons, respectively.

To test whether diazepam-induced potentiation of sIPSC frequency involved GABA_A_R-mediated excitation of VTA GABA neurons, we pharmacologically blocked excitatory GABA_A_R function. Previous studies reported that depolarizing current through GABA_A_R is mediated by the outward flow of HCO_3_^–^ ions, which becomes predominant during the loss of the hyperpolarizing Cl^−^ gradient (Staley et al., 1995; Laviolette et al., 2004; Doyon et al., 2016; Ostroumov et al., 2016). Given that the carbonic anhydrase inhibitor ACTZ blocks the intracellular production of bicarbonate ions and the depolarizing bicarbonate ion efflux, we hypothesized that ACTZ would prevent the diazepam-induced sIPSC frequency increase following nicotine exposure. Bath application of ACTZ (10 μM) did not change basal sIPSC frequency between control and nicotine groups, nor did it change control responses to diazepam. However, exposure to diazepam in the presence of ACTZ blocked the increase in sIPSC frequency after nicotine (Fig. 5*C*; 70.2 ± 7.0% in nicotine-treated animals, and 63.5 ± 6.3% in saline-treated controls, *n* = 7 cells/group, *n* = 3 rats/group, *t* = 0.710, *p* = 0.491; Table 1, line r), confirming that this increase was due, at least in part, to excitatory GABA_A_R function.

Next, we hypothesized that excitatory GABA_A_R function following nicotine exposure derived from the downregulation of KCC2 in VTA GABA neurons. Previous work demonstrated that enhancing Cl^−^ extrusion with the KCC2 agonist, CLP290, prevents GABAergic excitation of VTA GABA neurons in conditions of dysfunctional KCC2 (Gagnon et al., 2013; Ostroumov et al., 2016; Thomas et al., 2018). To determine whether CLP290 can prevent the enhanced GABA release onto DA neurons observed in the nicotine-pretreated animals, we measured sIPSC frequency in slices incubated in CLP290 (>1 h, 10 μM) prior to recordings. The nicotine-mediated potentiation of the sIPSC frequency elicited by diazepam was prevented by CLP290 incubation (Fig. 5*D*): 65.5 ± 5.9% in nicotine-pretreated animals (red bar with CLP290 treatment) and 74.9 ± 4.3% in saline-treated controls (black bar with CLP290 treatment, *n* = 7, *n* = 8 cells/group, *n* = 3, *n* = 4 rats/group, *t* = −1.253, *p* = 0.232; Table 1, line s). Taken together, these results indicate that the effect of acute nicotine pretreatment on diazepam-induced GABA network activity in the VTA was mediated by impaired Cl^−^ homeostasis and altered GABAergic circuit function.

### Acute nicotine attenuates diazepam-induced DA activity *in vivo*

**Figure 6.**
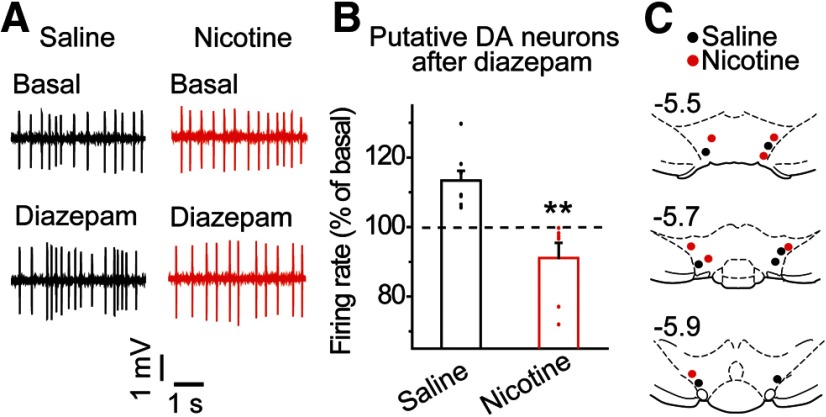
Acute nicotine exposure attenuates diazepam-induced VTA DA neuron firing rates *in vivo*. ***A***, Representative *in vivo* recordings from putative DA neurons before and after diazepam administration (dose, i.v.) in the nicotine (red) and saline (black) groups. ***B***, Diazepam increased the firing rate of putative DA neurons in the saline group (black) but failed to increase the firing rate of DA neurons in the nicotine group (red); **p < 0.01, significantly different by *t* test, *n* = 7 rats/group. ***C***, Recording sites of putative DA neurons in the VTA for nicotine-treated (red) and saline-treated (black) animals.

Given that benzodiazepine application typically increases VTA DA neuron firing through disinhibition (O'Brien and White, 1987; Tan et al., 2010), we hypothesized that nicotine-induced depolarization of E_GABA_ in VTA GABA neurons would attenuate diazepam-induced DA responses in the lateral VTA. To test this, we conducted *in vivo* single-unit recordings of lateral VTA DA neurons in anesthetized rats. DA neurons were identified based on their electrophysiological and pharmacological properties (see Materials and Methods; Fig. 1*G*,*H*). No significant difference in the mean basal firing rate between groups was observed, 8.0 ± 1.0 Hz after saline versus 8.2 ± 0.9 Hz after nicotine, *n* = 7 rats/group, *t* = 0.094, *p* = 0.926 (Table 1, line t). The spontaneous firing rates of VTA DA neurons were monitored before and after intravenous infusion of diazepam (0.25 mg/kg). The selected dose of diazepam was chosen because it disinhibits VTA DA neurons in rodents and extrapolates to a single dose of 2 mg in humans (O'Brien and White, 1987; Nair and Jacob, 2016). Diazepam administration induced a substantial increase in the spontaneous firing rate of lateral VTA DA neurons in saline-treated controls (Fig. 6*A*,*B*, black data): 113.4 ±3.2% of basal, *n* = 7 rats. In contrast, DA neurons from nicotine-pretreated animals did not show significant increases in DA firing (Fig. 6*A*,*B*, red data): 91.1 ± 4.3% of basal, *n* = 7 rats, *t* = −4.11 882, *p* = 0.001 (Table 1, line u). The VTA recording sites from saline-treated (black) and nicotine-treated (red) rats were similar across experiments (Fig. 6*C*). Taken together, our data indicate that acute nicotine exposure modified the pharmacological effects of diazepam on VTA circuitry, in particular serving to attenuate diazepam-induced DA responses which correlate with the increased sIPSC frequency observed in the *ex vivo* experiments.

### Acute nicotine increases diazepam intake

**Figure 7.**
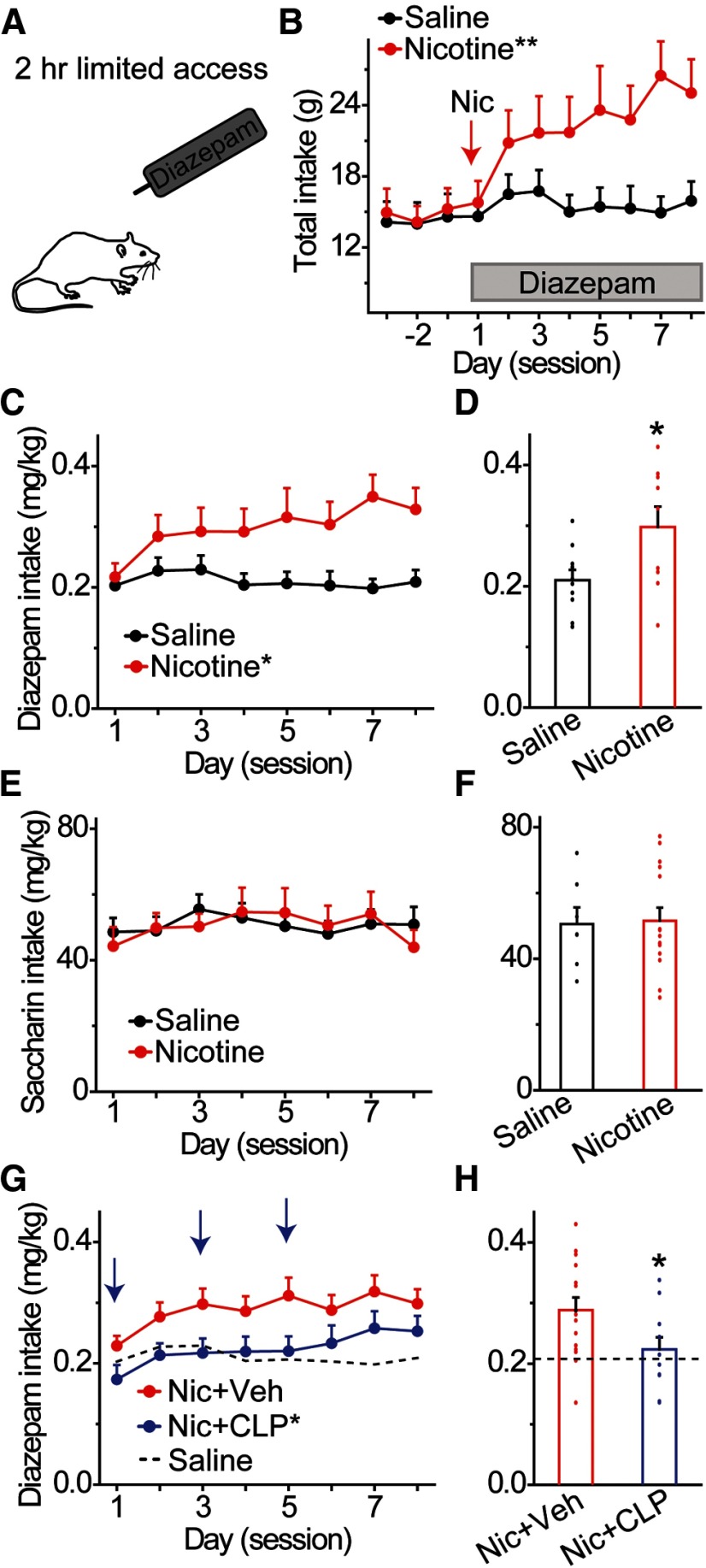
Increased diazepam consumption in nicotine-treated animals is reduced by KCC2 activation. ***A***, Animals consumed saccharin during daily 2-h sessions prior to adding diazepam in the drinking solution. Rats received an acute injection of nicotine or saline 7 h before the first diazepam drinking session. ***B***, Daily fluid intake was measured in saline and nicotine-treated rats. Nicotine-treated rats showed greater daily intake compared with saline-treated controls; **p < 0.01, significantly different by ANOVA with repeated measures. ***C***, Time course of mean daily diazepam intake for 8 d, following acute injection of nicotine (red) or saline (black); *p < 0.05, significantly different by ANOVA with repeated measures. ***D***, Rats receiving a single injection of nicotine (red) consumed significantly more diazepam (mg/kg) across 8 d than rats receiving a saline injection (black); *p < 0.05, significantly different from the control group by *t* test, *n* = 10 rats/group. ***E***, Effects of nicotine or saline pretreatment on daily saccharin intake (mg/kg), in the absence of diazepam. Nicotine-treated animals (red) showed similar saccharin intake compared with saline-treated controls (black) for 8 d after injection. ***F***, Effects of nicotine and saline pretreatment on mean saccharin intake. Rats receiving a single injection of nicotine (red) or saline (black) consumed similar amounts of saccharin (mg/kg) across 8 d. ***G***, Effects of CLP290 on daily diazepam intake. Vehicle (red data) or CLP290 (blue data) was injected intraperitoneally 45 min prior to diazepam drinking on non-consecutive days (blue arrows). Nicotine-pretreated animals injected with CLP290 showed significantly reduced diazepam consumption compared with nicotine-injected rats treated with vehicle. Diazepam consumption in saline-treated control rats is shown for comparison (dotted line); *p < 0.05, significantly different from the nicotine vehicle group by ANOVA with repeated measures. ***H***, Effects of CLP290 on mean diazepam intake over the 8-d drinking period. Animals pretreated with nicotine and CLP290 showed significantly reduced ethanol consumption compared with nicotine and vehicle-treated rats. Diazepam consumption in saline vehicle-treated control rats is shown for comparison (dotted horizontal line); *p < 0.05, significantly different from the nicotine vehicle group by *t* test, *n* = 11, *n* = 15 rats/group.

Previous work demonstrated that nicotine administration leads to increased consumption of ethanol (Doyon et al., 2013; Thomas et al., 2018). Ethanol and diazepam exert their actions on the mesolimbic DA system, in part, through activation of GABA_A_R on VTA GABA neurons. Therefore, we hypothesized that, like ethanol, diazepam consumption would be increased following acute nicotine exposure. To measure diazepam consumption, we used a 2-h limited access home cage drinking paradigm to elicit substantial short-term diazepam intake in rats, without causing the motor impairment produced by large diazepam doses that could confound interpretation of the results (Fig. 7*A*; Söderpalm et al., 1991). Stable consumption of saccharin (0.125%, w/v) was first established followed by the introduction of diazepam (0.005 mg/ml) into the drinking solution. This dose of diazepam was selected based on the volume of saccharin solution consumed to correspond with the dose of diazepam given for our in vivo DA neuron recordings. Animals receive an acute injection of either nicotine (0.4 mg/kg) or saline ∼7–8 h prior to the first diazepam drinking session. Upon adding diazepam to the drinking solution, nicotine-treated animals substantially increased total fluid intake compared with saline-treated controls (Fig. 7*B*): group × day, *F*_(10,170)_ = 6.12, *p* = 0.000 (Table 1, line v). Analysis of diazepam consumption according to individual animal weights revealed greater drug intake among nicotine-treated rats compared with controls (Fig. 7*C*): group, *F*_(1,17)_ = 5.72, *p* = 0.029 (Table 1, line w). Mean diazepam intake across the 8-d measurement period was significantly higher for the nicotine-treated group (0.30 ± 0.03 mg/kg) compared with the saline-treated group (0.21 ± 0.02 mg/kg; Fig. 7*D*, *n* = 9, *n* = 10 rats/group, *t* = 2.391, *p* = 0.029; Table 1, line x). In separate groups of animals, we measured saccharin consumption in the absence of diazepam and found no difference between nicotine and saline-treated rats (Fig. 7*E*): group, *F*_(1,20)_ = 0.06, *p* = 0.940 for daily intake (Table 1, line y). Mean saccharin intake across the 8 d was 50.8 ± 4.03 mg/kg for the nicotine-pretreated group and 50.3 ± 5.09 mg/kg for the saline pretreated group (Fig. 7*F*, *n* = 7, *n* = 15 rats/group, *t* = 0.077, *p* = 0.940; Table 1, line z). Therefore, the increased diazepam intake in nicotine-pretreated animals was not due to an increase in saccharin intake.

Given that the KCC2 agonist, CLP290, restored diazepam-induced VTA GABA signaling *ex vivo*, we hypothesized that enhancing Cl^−^ extrusion would also prevent elevated diazepam consumption following nicotine exposure. Nicotine-treated rats received systemic injections of CLP290 (10 mg/kg) or vehicle 45 min prior to diazepam access sessions on non-consecutive days (Fig. 7*G*, arrow). Compared with nicotine-treated rats that received injections of vehicle (Fig. 7*G*, red data), nicotine-treated rats that received injections of CLP290 significantly decreased daily diazepam consumption (Fig. 7*G*, blue data): group, *F*_(1,24)_ = 4.74, *p* = 0.040 (Table 1, line aa). Mean diazepam intake over the 8-d period was also significantly lower in nicotine-treated rats (Fig. 7*H*) that received injections of CLP290 (0.22 ± 0.02 mg/kg, blue bar) compared with nicotine-treated rats that received injections of vehicle (0.29 ± 0.02 mg/kg, red bar), *n* = 11, *n* = 15 rats/group, *t* = −2.176, *p* = 0.040 (Table 1, line bb). These data were indistinguishable from the saline-treated control group (Fig. 7*H*, dotted horizontal line).

## Discussion

In the present work, we demonstrated in rats that exposure to nicotine modifies the pharmacological action of diazepam on VTA circuitry and is associated with increased diazepam intake. In VTA GABA neurons, acute nicotine exposure caused a depolarizing shift in the GABA_A_R reversal potential (E_GABA_), which corresponded with diminished Cl^−^ extrusion capacity and decreased KCC2 expression. These nicotine-induced alterations in Cl^−^ homeostasis shifted the effect of diazepam on VTA GABA neurons from inhibition toward excitation and were associated with enhanced GABAergic inhibition of DA neurons in the lateral VTA on diazepam exposure. Critically, enhancing KCC2-mediated Cl^−^ extrusion in nicotine-treated animals prevented alterations in diazepam-induced VTA GABA transmission (Fig. 5*D*) and reduced diazepam consumption to control levels (Fig. 7*G*,*H*).

A depolarizing shift in E_GABA_ results in decreased synaptic inhibition or even paradoxical GABAergic excitation of VTA GABA neurons. Although previous work reports depolarizing shifts in GABA_A_R signaling within the VTA following repeated adolescent nicotine administration (Thomas et al., 2018), the current results demonstrate that these adaptations can arise in response to a single nicotine exposure in adult animals. Another important difference between chronic adolescent versus acute adult nicotine exposure is the duration of nicotine-induced neuroadaptations. Previous studies showed that dysregulation of VTA GABA signaling persists for more than three weeks following repeated nicotine in adolescence, whereas the effect of acute nicotine in adults wanes within several days (Doyon et al., 2013; Thomas et al., 2018). In addition to nicotine, treatments that shift GABA_A_R signaling toward excitation in VTA GABA neurons include abrupt withdrawal from chronic morphine or ethanol, as well as aversive or pain states (Taylor et al., 2015, 2016; Ostroumov et al., 2016; Nelson et al., 2018). Together with this literature, our findings indicate that experience-dependent depolarizing shifts in GABA_A_R signaling represent a common, yet remarkably understudied, form of inhibitory synaptic plasticity in the VTA.

Plasticity of inhibitory transmission on VTA GABA neurons can modify the pharmacological action of addictive drugs that target GABA_A_R (Vashchinkina et al., 2014). In agreement with previous studies (O'Brien and White, 1987; Tan et al., 2010), our findings show that positive modulation of GABA_A_R signaling by diazepam attenuates VTA GABA neuron activity in drug naïve animals. However, after nicotine exposure, diazepam-induced inhibition of VTA GABA neurons was diminished and shifted toward excitation. This transition in GABA_A_R function arises from impaired Cl^−^ extrusion and downregulation of the K^+^, Cl^−^ transporter KCC2 (Staley et al., 1995; Ostroumov et al., 2016). Upon diazepam-induced potentiation of GABA_A_R activity, diminished function of KCC2 leads to the accumulation of Cl^−^ inside the neuron and subsequent collapse of the Cl^−^ gradient (Fig. 2*D*,*E*), which is necessary for the hyperpolarizing GABA_A_R function (Staley et al., 1995; Hewitt et al., 2009). An activity-dependent decrease in the hyperpolarizing Cl^−^ gradient decreases GABAergic inhibition and unmasks an outward flux of HCO_3_^–^ ions through GABA_A_Rs, resulting in neuronal depolarization/excitation (Bormann et al., 1987; Kaila, 1994; Hewitt et al., 2009; Doyon et al., 2016). Consistent with this model, enhancing Cl^−^ extrusion with CLP290 or attenuating HCO_3_^–^ efflux with ACTZ prevented diazepam-induced excitation of VTA GABA circuitry (Fig. 5*B–D*).

Downstream of KCC2 and VTA GABA neuron alterations, we observed diazepam-induced inhibition of lateral VTA DA neurons and increased diazepam intake in nicotine-treated animals. We speculate that adaptations in the inhibitory input onto VTA DA neurons arise from local GABA neurons, but other afferent projections may play a role as well. The correlation between attenuated diazepam-induced DA responses and increased addictive behaviors is consistent across different species and multiple drugs of abuse, including alcohol, nicotine, cocaine and marijuana (Martinez et al., 2007; Lack et al., 2008; Audrain-McGovern et al., 2012; Wang et al., 2012; Doyon et al., 2013; Volkow et al., 2014; Twining et al., 2015; Ostroumov et al., 2016; Büchel et al., 2017; Thomas et al., 2018). Clinical and preclinical studies associate blunted DA signaling with decreased reward sensitivity and perpetuation of drug use as a means to overcome the attenuated subjective effects (Martinez et al., 2007; Volkow et al., 2010, 2014; Taylor et al., 2015, 2016; Twining et al., 2015). In parallel, GABA-mediated persistent decreases in DA neuron activity effectively increase phasic DA responses to salient, drug-related stimuli that may drive addictive-like behaviors (Wanat et al., 2009; Tolu et al., 2013; Morozova et al., 2016; Schindler et al., 2016; Kruse et al., 2017). In this way, phasic DA signals stand out from the background DA, producing an increased signal-to-noise for salient events. Our electrophysiological data were collected in slices or in anesthetized animals and correspond to pharmacological responses to diazepam in the absence of cues. Therefore, future work should examine how exposure to nicotine alters phasic and tonic DA responding *in vivo* during addictive behaviors.

Recent epidemiological studies report a steep rise in benzodiazepine misuse and overdose mortality, occurring most commonly in poly drug users (Dunbar et al., 1988; Woods et al., 1992; Griffiths and Weerts, 1997; Lekka et al., 1997; Neutel, 2005; Licata and Rowlett, 2008; Bachhuber et al., 2016; Lembke et al., 2018; Maust et al., 2019). We showed that nicotine pretreatment increases diazepam intake for several days. The prolonged behavioral effect of a single nicotine exposure suggests that KCC2 downregulation serves as a form of metaplasticity, facilitating downstream diazepam-induced neuroadaptations that maintain elevated consumption (Creed and Lüscher, 2013; Cui et al., 2013; Ostroumov and Dani, 2018). An important parameter in our experimental design is that the nicotine exposure was well separated (7–15 h) from diazepam exposure, which allowed us to examine the lasting neural circuit consequences of the treatment not the proximal effect of nicotine itself. Although dysregulated VTA GABA transmission was observed following repeated nicotine administration (Fig. 3; Thomas et al., 2018), future work should explore the impact of chronic nicotine-induced KCC2 downregulation on nicotine-benzodiazepine coabuse.

Following nicotine administration, elevated diazepam intake was associated with midbrain KCC2 dysfunction and could be prevented by the KCC2 agonist, CLP290. These results suggest that Cl^−^ extrusion enhancers may serve as a therapeutic strategy to mitigate excessive diazepam consumption in smoking populations (Dunbar et al., 1988; Lekka et al., 1997; Neutel, 2005). Moreover, KCC2 hypofunction and compromised GABAergic inhibition have been observed following other risk factors for benzodiazepine abuse, including opiates and alcohol (Laviolette et al., 2004; Taylor et al., 2016; Nelson et al., 2018), suggesting that alterations in midbrain KCC2 are a neurobiological mechanism contributing to the vulnerability to benzodiazepine abuse. Taken together, our results reveal that acute nicotine exposure induces long-lasting alterations in mesolimbic responses to benzodiazepines and promotes diazepam consumption in rats. While it is known that nicotine use increases the risk for abuse of other drugs, our data demonstrate that this association extends to benzodiazepines. Moreover, we provide novel insight into the circuit and molecular adaptations giving rise to heightened diazepam consumption after nicotine exposure.

## Synthesis

Reviewing Editor: Lorna Role, NINDS Decisions are customarily a result of the Reviewing Editor and the peer reviewers coming together and discussing their recommendations until a consensus is reached. When revisions are invited, a fact-based synthesis statement explaining their decision and outlining what is needed to prepare a revision will be listed below. The following reviewer(s) agreed to reveal their identity: Daniel McGehee, Thomas Hnasko.

The authors have investigated mechanisms by which acute nicotine can influence benzodiazepine’s effects on midbrain dopamine system in rats. Here they pretreated rats with nicotine (0.4mg/kg) or saline 15 hours prior to testing neuroadaptations, which included assessing changes in neuronal excitability and inhibitory transmission in the VTA, along with behavioral testing.

Overall the studies are quite well done, the conclusions drawn are thoughtful and sound, and this could represent an important addition to the literature. However, there are some conflicts with the prior literature that should be addressed, some changes and additions to the text and figures that should be incorporated and some aspects of the experimental work that could be extended to strengthen the manuscript. We encourage the authors to address these issues as outlined below.

1. Additional experimental work and/or discussion required

a. The data presented in Fig 1D and 1E are key to the author’s main conclusions regarding nicotine changes in KCC2, but they are using whole cell recording, which should clamp the Cl concentration gradient. Showing that pretreatment with a KCC2 inhibitor eliminates the difference seen in tissue from nicotine pretreated animals would strengthen their interpretation considerably. In any case, it is important to explain this result, and/ or repeat the experiment with grammicidin.

b. The authors report dramatic effects of their single nicotine injection, but they present the relevance of their observations in the context of chronic nicotine exposure. The use of a single nicotine injection here is not sufficient to make that important link to nicotine-benzodiazepine co-abuse. Although testing for potential differences in effect duration would strengthen the study considerably, the authors should at least discuss the limitations of their data if no additional experiments are done.

c. The authors should explain why they are seeing effects following a single nicotine exposure in adult animals, given that these were not reported previously Specifically, Thomas et al (2018) reported effects of chronic nicotine exposure in adolescents, but did not see the same persistent effects in adult animals.

d. It is not obvious why nicotine exposed mice, where diazepam inhibits rather than excites DA neurons, should consume more of it as they do. If the authors accept the conclusions of Tan et al, that diazepam is reinforcing because it increases DA neuron activity, then why would a treatment that reverses this effect of diazepam increase diazepam self administration? Discuss?

e. Demonstration that GABA reversal potential in DA neurons is unaffected by nicotine in Figure 1, or that non-DA neurons recorded in VTA show diazepam-induced increase in firing after nicotine would strengthen the conclusions

f. The authors define how they use ‘GABA’ and ‘DA’ neurons in the methods. While others may disagree with the utility of these criterion they are reasonably defined and defended here. However, the authors should consider the possibility that some of the non-DA neurons are glutamate releasing. While these glutamate cells are not all that abundant in lateral VTA, the authors do not precisely define how they identify lateral VTA in their ex vivo recordings. And several studies show that VTA glutamate neurons cells represent a sizable proportion of the VTA projections to frontal cortex and NAc, and that they express nicotinic ACh receptors (Yamaguchi 2011, Hnasko 2012, Taylor 2014, Yan/Drenan 2018).

2. Additions/ modifications to text and figures:

a. Statistics and software:

- Software used for IPSC analysis, firing rate and statistical comparisons were not stated and should be included in the revised manuscript

- The use of a paired t-test is not appropriate here, as these measures were not obtained from the same animals - i.e. nicotine and saline injections occurred in different populations of rats.

b. Clarification/ Modifications to figures/ methods

-Figure 1D and 1E - The Y-axis label needs correcting, the values are % of baseline IPSC amplitudes relative to the mean of stimulations 1-5. The description of the data in the legend is confusing and incomplete in its present form. The n value for D is not presented and the n value in E is not clear n=8 rats? And ‘16 cells’ refers to which groups? Were the same neurons tested under each of the different voltage conditions? The labels for the Vm conditions should be moved to the top of each panel in 1D and 1E for clarity.

-In general the number of mice should be reported in addition to the number of cells recorded.

-It’s not clear why CLP290 was given every other day in the final behavioral experiment, or if the first injection was given after or concurrently with nicotine. Adding a bit of detail and rationale for this scheme to the results would be helpful.

c. Modifications to text:

-There appears to be very different effects of puff application of diazepam vs bath application as shown in Figure 2. This should be addressed in the text.

-The use of the CA inhibitor ACTZ in the experiments presented in Figure 3,, needs a better introduction -- it should be made clear to the reader how CA inhibition will affect the Cl gradients. The pretreatment times with this drug and CLP should also be presented clearly in the text.

-Pg 18 line 350, the authors use the word ‘potentiation’, but ‘increase’ is more accurate description of the result.

-The spontaneous amplitude data is missing from lines 353-54.

-The data point to a role for local GABA neurons in VTA, but don’t rule out a role for distal GABA inputs to VTA as well. This could be noted in discussion.

## Author Response

We have performed new experiments and revised the manuscript to address the criticisms raised in the initial review. New data panels are presented throughout the main text highlighted in yellow and 2 new multi-panel figures cover the experimental requests. We addressed each of the reviewers’ comments below.

1. Additional experimental work and/or discussion required

a. The data presented in Fig 1D and 1E are key to the author’s main conclusions regarding nicotine changes in KCC2, but they are using whole cell recording, which should clamp the Cl concentration gradient. Showing that pretreatment with a KCC2 inhibitor eliminates the difference seen in tissue from nicotine pretreated animals would strengthen their interpretation considerably. In any case, it is important to explain this result, and/or repeat the experiment with gramicidin.

We have provided additional explanation for the experiment in Fig 2D and 2E (former Fig 1D and 1E), which probes the efficacy of Cl- extrusion and has been used by many different labs (Hewitt et al., 2009; Ferrini et al., 2013; Ostroumov et al., 2016; Thomas et al., 2018). In brief, we used the whole-cell configuration to establish a similar, basal Cl- gradient in cells from nicotine and saline-treated rats. Under these conditions, collapse of the experimenter-set Cl- gradient becomes readily apparent during trains of evoked IPSCs. When holding the cell at a membrane potential that favors Cl- influx (0 mV), KCC2 must rapidly extrude Cl- to maintain the established Cl- gradient, which is reflected in the large amplitudes of evoked IPSCs (Fig 2E, black data). After nicotine exposure, KCC2 fails to maintain the established Cl- gradient, and the progressive decrease in the evoked IPSC amplitudes (Fig 2E, red data) indicates Cl- accumulation inside the cell. These results complement the gramicidin-perforated patch-clamp assessment of KCC2 function shown in Figs 2B and 2C. Here, we preserve the endogenous intracellular Cl- concentration and measure the intact Cl- gradient in GABA neurons from each group (Figs 2B, 2C). Taken together with the direct measurements of transporter protein expression (Fig 2F and 2G), our results in Fig 2 strongly implicate KCC2 impairment following nicotine exposure.

b. The authors report dramatic effects of their single nicotine injection, but they present the relevance of their observations in the context of chronic nicotine exposure. The use of a single nicotine injection here is not sufficient to make that important link to nicotine-benzodiazepine co-abuse. Although testing for potential differences in effect duration would strengthen the study considerably, the authors should at least discuss the limitations of their data if no additional experiments are done.

We agree that a single experimenter-induced nicotine exposure has limited relevance to chronic nicotine abuse. To address this issue, we performed additional new experiments measuring chloride homeostasis in rats that received 1-hour/day (for 7d) access to nicotine in saccharin-sweetened drinking solution (new Figs 3A and 3B). We observed that 7 consecutive nicotine drinking sessions impaired chloride homeostasis and reduced KCC2 expression in the VTA when measured after the last nicotine drinking day (new Figs 3C and 3D). These results indicate that acute experimenter-induced exposure and repeated volitional nicotine administration produce comparable adaptations that contribute to increased diazepam intake. Nevertheless, we acknowledge that future work is needed to explore the impact of chronic nicotine exposure on nicotine-benzodiazepine co-abuse, and we have revised Discussion to make these issues clear.

c. The authors should explain why they are seeing effects following a single nicotine exposure in adult animals, given that these were not reported previously Specifically, Thomas et al (2018) reported effects of chronic nicotine exposure in adolescents, but did not see the same persistent effects in adult animals.

Our new results, using a different experimental design, are consistent with the results of Thomas et al (2018). We have emphasized the differences between our study and previous literature in the revised Discussion section. Specifically, Thomas et al (2018) made their measurements 4 weeks after the nicotine treatment, and they did not observe nicotine-induced dysfunction of VTA GABA transmission in adult rats when measured at 4 weeks after nicotine exposure in adulthood (see Fig 2 in Thomas et al., 2018). However, when the same group examined nicotine-induced VTA circuitry adaptations just a few days after nicotine exposure in adulthood (Doyon et al., 2013), then they did observe changes like those we see. Therefore, our findings fully agree with the literature, indicating that single experimenter-administered nicotine readily, but reversibly, impairs inhibitory transmission in the VTA in adulthood for a few days. Thomas et al showed that these changes are more long lasting when the chronic nicotine exposures occur during adolescence.

d. It is not obvious why nicotine exposed mice, where diazepam inhibits rather than excites DA neurons, should consume more of it as they do. If the authors accept the conclusions of Tan et al, that diazepam is reinforcing because it increases DA neuron activity, then why would a treatment that reverses this effect of diazepam increase diazepam self administration? Discuss?

We have addressed these seemingly contradictory results in the Discussion. The correlation between attenuated diazepam-induced dopamine responses and increased addictive behaviors is consistent across different species and multiple drugs of abuse, including alcohol, nicotine, cocaine and marijuana (Martinez et al., 2007; Lack et al., 2008; Audrain-McGovern et al., 2012; Wang et al., 2012; Doyon et al., 2013; Volkow et al., 2014; Twining et al., 2015; Ostroumov et al., 2016; Buchel et al., 2017; Thomas et al., 2018). Clinical and preclinical studies associate blunted dopamine signaling with decreased reward sensitivity and perpetuation of drug use as a means to overcome the attenuated subjective effects (Martinez et al., 2007; Volkow et al., 2010; Volkow et al., 2014; Taylor et al., 2015; Twining et al., 2015; Taylor et al., 2016). In parallel (and likely more importantly), GABA-mediated persistent decreases in DA neuron activity effectively increase phasic DA responses to salient, drug-related stimuli that may drive addictive-like behaviors (Wanat et al., 2009; Tolu et al., 2013; Morozova et al., 2016; Schindler et al., 2016; Kruse et al., 2017). In this way, phasic DA signals stand out from the background DA, producing an increased signal-to-noise for salient events. Our electrophysiological data were collected in slices or in anesthetized animals and correspond to pharmacological responses to diazepam in the absence of cues. Therefore, future work should examine how exposure to nicotine alters phasic and tonic DA signaling in vivo during addictive behaviors.

e. Demonstration that GABA reversal potential in DA neurons is unaffected by nicotine in Figure 1, or that non-DA neurons recorded in VTA show diazepam-induced increase in firing after nicotine would strengthen the conclusions

We agree it is important to show the diazepam-induced increase in non-DA neuron firing following nicotine exposure. Our new Figs 4B and 4C demonstrate that putative VTA GABA neurons from nicotine-treated rats increase their firing rate in response to diazepam. Previous work has demonstrated that the GABA reversal potential in DA neurons is unaffected by nicotine (Thomas et al., 2018). Furthermore, it has been previously shown in multiple publications that DA neurons within the VTA and substantia nigra lack expression for KCC2 (Gulacsi et al., 2003; Taylor et al., 2015; Ostroumov et al., 2016; Taylor et al., 2016; Thomas et al., 2018), which is consistent with the presence of another chloride extrusion mechanism in DA neurons.

f. The authors define how they use ‘GABA’ and ‘DA’ neurons in the methods. While others may disagree with the utility of these criterion they are reasonably defined and defended here. However, the authors should consider the possibility that some of the non-DA neurons are glutamate releasing. While these glutamate cells are not all that abundant in lateral VTA, the authors do not precisely define how they identify lateral VTA in their ex vivo recordings. And several studies show that VTA glutamate neurons cells represent a sizable proportion of the VTA projections to frontal cortex and NAc, and that they express nicotinic ACh receptors (Yamaguchi 2011, Hnasko 2012, Taylor 2014, Yan/Drenan 2018).

We agree that VTA neuron identification requires careful consideration, and we now provide new data and new Fig 1 to address the reviewer’s concerns and clarify our identification criteria. As recommended, new Fig 1A provides a diagram of the area defined as the lateral VTA. The lateral VTA consists predominantly of dopamine and GABA neurons, as previous studies demonstrate that glutamatergic neurons are mostly located in rostral and medial parts of the VTA (Kawano et al., 2006; Yamaguchi et al., 2007; Nair-Roberts et al., 2008; Yamaguchi et al., 2011; Faget et al., 2016). Nevertheless, we now used additional immunohistochemical and pharmacological approaches to enhance accurate identification of lateral VTA neurons in both ex vivo and in vivo preparations (new Figs 1C, 1F, and 1H). The combination of conservative electrophysiological, molecular, and pharmacological criteria remains the most reliable methodology to identify DA neurons in the lateral VTA in non-transgenic rodents (Margolis et al., 2006; Zhang et al., 2010; Lammel et al., 2011; Li et al., 2012; Ungless and Grace, 2012; Eddine et al., 2015).

2. Additions/ modifications to text and figures:

a. Statistics and software:

- Software used for IPSC analysis, firing rate and statistical comparisons were not stated and should be included in the revised manuscript

This information is now included in the manuscript.

- The use of a paired t-test is not appropriate here, as these measures were not obtained from the same animals - i.e. nicotine and saline injections occurred in different populations of rats.

We agree and we utilized a paired t-test only for the western blot experiments, where each pair of nicotine and saline-treated rats was prepared independently and run on separate gels. Specifically, each rat within a pair of littermates was randomly assigned to either the nicotine or saline group. They were then housed together, and this pair underwent treatments and VTA harvesting on the same day. Protein samples from rats within one pair were always run on the same gel. In contrast, different pairs were treated on different days and run on separate gels. We observed a high variability between separate gels but the normalized KCC2 signal from nicotine-treated rats was always lower when compared to its paired saline-treated littermate. For these reasons, our experimental design for western blot analysis is appropriately analyzed with a paired t-test.

b. Clarification/ Modifications to figures/ methods

-Figure 1D and 1E - The Y-axis label needs correcting, the values are % of baseline IPSC amplitudes relative to the mean of stimulations 1-5. The description of the data in the legend is confusing and incomplete in its present form. The n value for D is not presented and the n value in E is not clear n=8 rats? And ‘16 cells’ refers to which groups? Were the same neurons tested under each of the different voltage conditions? The labels for the Vm conditions should be moved to the top of each panel in 1D and 1E for clarity.

We have provided all the requested modifications to the figures and legends. As the reviewer expected, for Figs 2D and 2E (former Figs 1D, 1E) the same neurons were tested under each voltage condition and the n values were 16 cells for the saline group and 8 cells for the nicotine group.

-In general the number of mice should be reported in addition to the number of cells recorded.

We have included the number of animals, along with the number of cells recorded, for each experiment in the revised manuscript.

-It’s not clear why CLP290 was given every other day in the final behavioral experiment, or if the first injection was given after or concurrently with nicotine. Adding a bit of detail and rationale for this scheme to the results would be helpful.

We have added the requested information to the Materials and Methods section. In our CLP290 experiments, we followed a previously published protocol (Thomas et al., 2018) that utilizes every other day administration of CLP290 because that study and our preliminary investigations indicated that CLP290 was effective for ∼2d. Therefore, CLP290 worked effectively when we injected every other day. The first injection occurs 45 mins prior to the initial diazepam exposure in the saccharin-sweetened drinking solution and approximately 7 hours after the acute exposure to nicotine.

c. Modifications to text:

-There appears to be very different effects of puff application of diazepam vs bath application as shown in Figure 2. This should be addressed in the text.

We believe that Fig 4 (former Fig 2) shows qualitatively similar effects of bath and local diazepam application. In saline-treated animals, both types of diazepam administration reduced the firing rate of VTA GABA neurons. Only after nicotine treatment did a puff application of diazepam produce less inhibition and even cause paradoxical excitation in a fraction of GABA neurons. On average, the effect of puff administration of diazepam produced a slight increase in GABA neuron activity following nicotine exposure, which was qualitatively similar to bath-application of diazepam. Any minor differences in the effects of puff application versus bath application of diazepam are likely due to the differing spatial and temporal delivery of diazepam in these two recording configurations. A puff application of diazepam results in a relatively fast, local administration of diazepam to the recording site, while bath application of diazepam produces a slower more diffuse administration of the drug to the entire slice. These distinctions may explain the minor differences in the observed effects, but in general our results show qualitatively similar effects of bath and local diazepam application on VTA GABA neuron firing rate.

-The use of the CA inhibitor ACTZ in the experiments presented in Figure 3, needs a better introduction -- it should be made clear to the reader how CA inhibition will affect the Cl gradients. The pretreatment times with this drug and CLP should also be presented clearly in the text.

We have included the requested information in the revised manuscript. Diminished function of KCC2 leads to the accumulation of Cl- inside the cell and a subsequent loss of the Cl- gradient. The loss of the hyperpolarizing Cl- gradient unmasks an outward flux of bicarbonate ions through GABAA receptors, resulting in neuronal depolarization/excitation (Staley et al., 1995; Doyon et al., 2016). The carbonic anhydrase inhibitor acetazolamide blocks the intracellular production of bicarbonate ions and the depolarizing bicarbonate ion efflux thereby preventing the excitatory shift in GABAA receptor function following nicotine exposure (Laviolette et al., 2004; Ostroumov et al., 2016).

-Pg 18 line 350, the authors use the word ‘potentiation’, but ‘increase’ is more accurate description of the result.

As recommended, we have changed the word to ‘increase’.

-The spontaneous amplitude data is missing from lines 353-54.

This information has been added.

-The data point to a role for local GABA neurons in VTA, but don’t rule out a role for distal GABA inputs to VTA as well. This could be noted in discussion.

The note has been added to the Discussion section.

REFERENCES

Audrain-McGovern J, Rodriguez D, Leventhal AM, Cuevas J, Rodgers K, Sass J (2012) Where is the pleasure in that? Low hedonic capacity predicts smoking onset and escalation. Nicotine Tob Res 14:1187-1196.

Buchel C et al. (2017) Blunted ventral striatal responses to anticipated rewards foreshadow problematic drug use in novelty-seeking adolescents. Nat Commun 8:14140.

Doyon N, Vinay L, Prescott SA, De Koninck Y (2016) Chloride Regulation: A Dynamic Equilibrium Crucial for Synaptic Inhibition. Neuron 89:1157-1172.

Doyon WM, Dong Y, Ostroumov A, Thomas AM, Zhang TA, Dani JA (2013) Nicotine decreases ethanol-induced dopamine signaling and increases self-administration via stress hormones. Neuron 79:530-540.

Eddine R, Valverde S, Tolu S, Dautan D, Hay A, Morel C, Cui Y, Lambolez B, Venance L, Marti F, Faure P (2015) A concurrent excitation and inhibition of dopaminergic subpopulations in response to nicotine. Sci Rep 5:8184.

Faget L, Osakada F, Duan J, Ressler R, Johnson AB, Proudfoot JA, Yoo JH, Callaway EM, Hnasko TS (2016) Afferent Inputs to Neurotransmitter-Defined Cell Types in the Ventral Tegmental Area. Cell Rep 15:2796-2808.

Ferrini F, Trang T, Mattioli TA, Laffray S, Del’Guidice T, Lorenzo LE, Castonguay A, Doyon N, Zhang W, Godin AG, Mohr D, Beggs S, Vandal K, Beaulieu JM, Cahill CM, Salter MW, De Koninck Y (2013) Morphine hyperalgesia gated through microglia-mediated disruption of neuronal Cl(-) homeostasis. Nat Neurosci 16:183-192.

Gulacsi A, Lee CR, Sik A, Viitanen T, Kaila K, Tepper JM, Freund TF (2003) Cell type-specific differences in chloride-regulatory mechanisms and GABA(A) receptor-mediated inhibition in rat substantia nigra. J Neurosci 23:8237-8246.

Hewitt SA, Wamsteeker JI, Kurz EU, Bains JS (2009) Altered chloride homeostasis removes synaptic inhibitory constraint of the stress axis. Nat Neurosci 12:438-443.

Kawano M, Kawasaki A, Sakata-Haga H, Fukui Y, Kawano H, Nogami H, Hisano S (2006) Particular subpopulations of midbrain and hypothalamic dopamine neurons express vesicular glutamate transporter 2 in the rat brain. J Comp Neurol 498:581-592.

Kruse LC, Schindler AG, Williams RG, Weber SJ, Clark JJ (2017) Maladaptive Decision Making in Adults with a History of Adolescent Alcohol use, in a Preclinical Model, Is Attributable to the Compromised Assignment of Incentive Value during Stimulus-Reward Learning. Front Behav Neurosci 11:134.

Lack CM, Jones SR, Roberts DC (2008) Increased breakpoints on a progressive ratio schedule reinforced by IV cocaine are associated with reduced locomotor activation and reduced dopamine efflux in nucleus accumbens shell in rats. Psychopharmacology (Berl) 195:517-525.

Lammel S, Ion DI, Roeper J, Malenka RC (2011) Projection-specific modulation of dopamine neuron synapses by aversive and rewarding stimuli. Neuron 70:855-862.

Laviolette SR, Gallegos RA, Henriksen SJ, van der Kooy D (2004) Opiate state controls bi-directional reward signaling via GABAA receptors in the ventral tegmental area. Nat Neurosci 7:160-169.

Li W, Doyon WM, Dani JA (2012) Quantitative unit classification of ventral tegmental area neurons in vivo. J Neurophysiol 107:2808-2820.

Margolis EB, Lock H, Hjelmstad GO, Fields HL (2006) The ventral tegmental area revisited: is there an electrophysiological marker for dopaminergic neurons? J Physiol 577:907-924.

Martinez D, Narendran R, Foltin RW, Slifstein M, Hwang DR, Broft A, Huang Y, Cooper TB, Fischman MW, Kleber HD, Laruelle M (2007) Amphetamine-induced dopamine release: markedly blunted in cocaine dependence and predictive of the choice to self-administer cocaine. Am J Psychiatry 164:622-629.

Morozova EO, Myroshnychenko M, Zakharov D, di Volo M, Gutkin B, Lapish CC, Kuznetsov A (2016) Contribution of synchronized GABAergic neurons to dopaminergic neuron firing and bursting. J Neurophysiol 116:1900-1923.

Nair-Roberts RG, Chatelain-Badie SD, Benson E, White-Cooper H, Bolam JP, Ungless MA (2008) Stereological estimates of dopaminergic, GABAergic and glutamatergic neurons in the ventral tegmental area, substantia nigra and retrorubral field in the rat. Neuroscience 152:1024-1031.

Ostroumov A, Thomas AM, Kimmey BA, Karsch JS, Doyon WM, Dani JA (2016) Stress Increases Ethanol Self-Administration via a Shift toward Excitatory GABA Signaling in the Ventral Tegmental Area. Neuron 92:493-504.

Schindler AG, Soden ME, Zweifel LS, Clark JJ (2016) Reversal of Alcohol-Induced Dysregulation in Dopamine Network Dynamics May Rescue Maladaptive Decision-making. J Neurosci 36:3698-3708.

Staley KJ, Soldo BL, Proctor WR (1995) Ionic mechanisms of neuronal excitation by inhibitory GABAA receptors. Science 269:977-981.

Taylor AM, Castonguay A, Taylor AJ, Murphy NP, Ghogha A, Cook C, Xue L, Olmstead MC, De Koninck Y, Evans CJ, Cahill CM (2015) Microglia disrupt mesolimbic reward circuitry in chronic pain. J Neurosci 35:8442-8450.

Taylor AM, Castonguay A, Ghogha A, Vayssiere P, Pradhan AA, Xue L, Mehrabani S, Wu J, Levitt P, Olmstead MC, De Koninck Y, Evans CJ, Cahill CM (2016) Neuroimmune Regulation of GABAergic Neurons Within the Ventral Tegmental Area During Withdrawal from Chronic Morphine. Neuropsychopharmacology 41:949-959.

Thomas AM, Ostroumov A, Kimmey BA, Taormina MB, Holden WM, Kim K, Brown-Mangum T, Dani JA (2018) Adolescent Nicotine Exposure Alters GABAA Receptor Signaling in the Ventral Tegmental Area and Increases Adult Ethanol Self-Administration. Cell Rep 23:68-77.

Tolu S, Eddine R, Marti F, David V, Graupner M, Pons S, Baudonnat M, Husson M, Besson M, Reperant C, Zemdegs J, Pages C, Hay YA, Lambolez B, Caboche J, Gutkin B, Gardier AM, Changeux JP, Faure P, Maskos U (2013) Co-activation of VTA DA and GABA neurons mediates nicotine reinforcement. Mol Psychiatry 18:382-393.

Twining RC, Wheeler DS, Ebben AL, Jacobsen AJ, Robble MA, Mantsch JR, Wheeler RA (2015) Aversive stimuli drive drug seeking in a state of low dopamine tone. Biol Psychiatry 77:895-902.

Ungless MA, Grace AA (2012) Are you or aren’t you? Challenges associated with physiologically identifying dopamine neurons. Trends Neurosci 35:422-430.

Volkow ND, Wang GJ, Fowler JS, Tomasi D, Telang F, Baler R (2010) Addiction: decreased reward sensitivity and increased expectation sensitivity conspire to overwhelm the brain’s control circuit. Bioessays 32:748-755.

Volkow ND, Wang GJ, Telang F, Fowler JS, Alexoff D, Logan J, Jayne M, Wong C, Tomasi D (2014) Decreased dopamine brain reactivity in marijuana abusers is associated with negative emotionality and addiction severity. Proc Natl Acad Sci U S A 111:E3149-3156.

Wanat MJ, Willuhn I, Clark JJ, Phillips PE (2009) Phasic dopamine release in appetitive behaviors and drug addiction. Curr Drug Abuse Rev 2:195-213.

Wang GJ, Smith L, Volkow ND, Telang F, Logan J, Tomasi D, Wong CT, Hoffman W, Jayne M, Alia-Klein N, Thanos P, Fowler JS (2012) Decreased dopamine activity predicts relapse in methamphetamine abusers. Mol Psychiatry 17:918-925.

Yamaguchi T, Sheen W, Morales M (2007) Glutamatergic neurons are present in the rat ventral tegmental area. Eur J Neurosci 25:106-118.

Yamaguchi T, Wang HL, Li X, Ng TH, Morales M (2011) Mesocorticolimbic glutamatergic pathway. J Neurosci 31:8476-8490.

Zhang TA, Placzek AN, Dani JA (2010) In vitro identification and electrophysiological characterization of dopamine neurons in the ventral tegmental area. Neuropharmacology 59:431-436.
